# A New *Neosuidasia* Species (Sarcoptiformes: Suidasiidae) from The Netherlands: Life Stage Morphology †

**DOI:** 10.3390/insects16090896

**Published:** 2025-08-27

**Authors:** Qing-Hai Fan, Farid Faraji

**Affiliations:** 1 Plant Health and Environment Laboratory, Ministry for Primary Industries, Auckland 1072, New Zealand; 2 Eurofins MITOX BV, Science Park 408, 1098 XH Amsterdam, The Netherlands; farid.faraji@as.eurofinseu.com; 3 Institute for Biodiversity and Ecosystem Dynamics, Section Population Biology, University of Amsterdam, 1098 XH Amsterdam, The Netherlands

**Keywords:** Acari, Astigmata, Acaroidea, Northwestern Europe, Holland, stored product mite, new species

## Abstract

The family Suidasiidae Hughes (Acari: Sarcoptiformes: Acaroidea) comprises a small but ecologically diverse group of mites. The genus *Neosuidasia* is currently monotypic, containing only the species *Neosuidasia faini* Ranganath & ChannaBasavanna. Although this genus exhibits a wide geographic distribution, ontogenetic research into it remains scarce. This study describes a new species, *Neosuidasia sjorsvandenbergi* sp. nov., found in domestic poultry in the Netherlands and comprehensively presents the morphological changes across all known life stages, including the adult female, male, tritonymph, protonymph, larva, and egg stages. By documenting the developmental changes and setal patterns throughout ontogeny, this study addresses a significant gap in the understanding of suidasiid mite development.

## Simple Summary

The family Suidasiidae consists of 18 free-living mite species distributed across seven genera, inhabiting a diverse array of habitats such as house dust, stored products, bird and insect nests, and even human tissues. Some species have adapted to life deep in sand dunes. The genus *Neosuidasia* includes a single species, originally found in poultry feed in India and later reported in Senegal, the Dominican Republic, and Mexico. Previous studies were focused largely on adult mites, with the immature stages remaining poorly understood. This study provides a comprehensive description of the adult female, adult male, tritonymph, protonymph, larva, and egg stages of a new species from a poultry farm in the Netherlands, offering new insights into its morphology and geographic distribution.

## 1. Introduction

Suidasiidae Hughes (Acari: Sarcoptiformes: Acaroidea) is a relatively small family comprising 18 free-living species from seven genera [1]. The group was originally established by Hughes (1948) as a subfamily, Suidasiinae, in the family Acaridae, with *Suidasia* designated as the type genus [2]. Fain and Philips (1978) later proposed the same subfamily independently, unaware of Hughes’s earlier designation [3]. O’Connor (1982) subsequently elevated Suidasiinae to family rank as Suidasiidae, redefining its morphological boundaries [4]. This elevation was later supported by Fain et al. (1993) [5] and reaffirmed by O’Connor (2009) in his comprehensive review of astigmatid families [6].

This family exhibits considerable morphological diversity and inhabits a wide ecological range. Its members have been found in diverse environments, including stored products and house dust [6,7]; whale meat infested with dermestid beetles [7]; bird nests and skins [6,7]; bat roosts [6]; insect nests [6,7]; and even human skin, as well as in the ears, intestines, and pulmonary system [8]. Notably, a few species are specialized in inhabiting the deep layers of sand dunes [5].

*Neosuidasia* was established based primarily on the following characters [9]: having an idiosomal cuticle that is neither wrinkled nor scale-like; fine external vertical setae (*ve*) located posterior to the internal vertical setae (*vi*); and external scapular setae (*sce*) slightly longer than the internal scapular setae (*sci*). The idiosomal setae are smooth. Males possess a pair of para-anal suckers, although these are not highly sclerotised. The apex of tarsus I lacks a dorsal spine but bears three prominent ventral spines. The solenidia *ω1* on tarsus I and *ω* on tarsus II are similar in form. The genus *Neosuidasia* currently consists of a single species, *Neosuidasia faini* Ranganath et ChannaBasavanna, 1983. It was originally described from poultry feed in Bangalore, India [9], and later reported in a woodlouse breeding facility in Dakar, Senegal [10], as well as in pig and poultry feed in the Dominican Republic and Mexico, intercepted in Cuba [11].

Early taxonomic work on suidasiid mites largely centered on their adult morphology, with minimal focus on the immature stages. Ontogenetic research remains especially limited for *Neosuidasia*, where such studies do not exist, and data for other genera are also sparse [5,12,13]. Manson (1973) [12] documented measurements for the larval stage of *Suidasia reticulata*, while Fain & Philips (1978) [13] outlined distinguishing characteristics of the protonymph, deutonymph, and tritonymph of *Sapracarus tuberculatus*. Similarly, Fain et al. (1993) [5] described distinguishing characteristics of the larva, protonymph, and tritonymph in *Namihacarus sahulosus*. Balmes-Pacia & Corpuz-Raros (1998) [14] added body size data for all life stages of *Suidasia pontifica*. These efforts highlight the utility of discrete morphological characters in differentiating the immature stages from adults. Nevertheless, in-depth analyses of setal development and transformations throughout ontogeny remain underrepresented in the literature.

The present study aims to describe a new species of *Neosuidasia* collected from a domestic poultry in the Netherlands based on the morphology of its life stages, including the adult female, male, tritonymph, protonymph, larva, and egg stages. This work contributes to a broader understanding of the morphological diversity and geographical distribution of the genus.

## 2. Materials and Methods

Mite specimens were collected from a laboratory culture originally established using samples collected from an open-cage home poultry in Monster, the Netherlands (52°02′18.6″ N, 4°12′27.4″ E). They were slide-mounted in modified Hoyer’s medium [15] and dried on a hotplate at a constant temperature of 70 °C. After drying, the slides were sealed with Glyptal (Glyptal Inc., Chelsea, MI, USA). The specimens were examined and illustrated using a drawing tube attached to a Nikon Eclipse 80i interference-phase contrast microscope (Nikon Corporation, Tokyo, Japan). Key morphological structures were re-examined for confirmation using a Zeiss Axio Imager 2 microscope (Carl Zeiss Microscopy, Oberkochen, Germany). Images were captured using a Zeiss AxioCam HRc camera (Carl Zeiss Microscopy, Oberkochen, Germany), montaged using Helicon Focus (Helicon Soft Ltd., Kharkiv, Ukraine), and edited in Adobe Photoshop 2023 (Adobe Inc., San Jose, CA, USA).

The idiosoma length was measured from the anterior margin to the posterior margin, and its width was recorded at the widest point. The setae were measured from the alveolus to the tip. Legs were measured from the base of the trochanter to the tip of the claw, with each leg segment measured from base to tip, excluding the pretarsus in the tarsus measurement. For adult females, measurements are provided for the holotype, followed by the range observed in the measured specimens in parentheses. Ranges are also provided for paratype males, tritonymphs, protonymphs, and larvae. All measurements are in micrometers (μm). The terminology for idiosomal chaetotaxy follows the system proposed by Griffiths et al. [16] and modified by Norton [17], while the palpal and leg chaetotaxy terminology is based on Grandjean [18,19], respectively. The terminology for the copulatory organ follows Witaliński et al. [20], with comparative terms from Klimov and O’Connor [21] provided in parentheses.

## 3. Systematics

### 3.1. *Suidasiidae Hughes*

Suidasiinae Hughes 1948: 550; Fain et Philips, 1978: 115.
Suidasiidae Hughes; OConnor, 1982: 155; Fain et al., 1993: 150; OConnor, 2009: 580.

### 3.2. Neosuidasia *Ranganath and ChannaBasavanna*

*Neosuidasia* Ranganath and ChannaBasavanna, 1983: 63.

Type species: *Neosuidasia faini* Ranganath and ChannaBasavanna, 1983.

**Figure 1:**
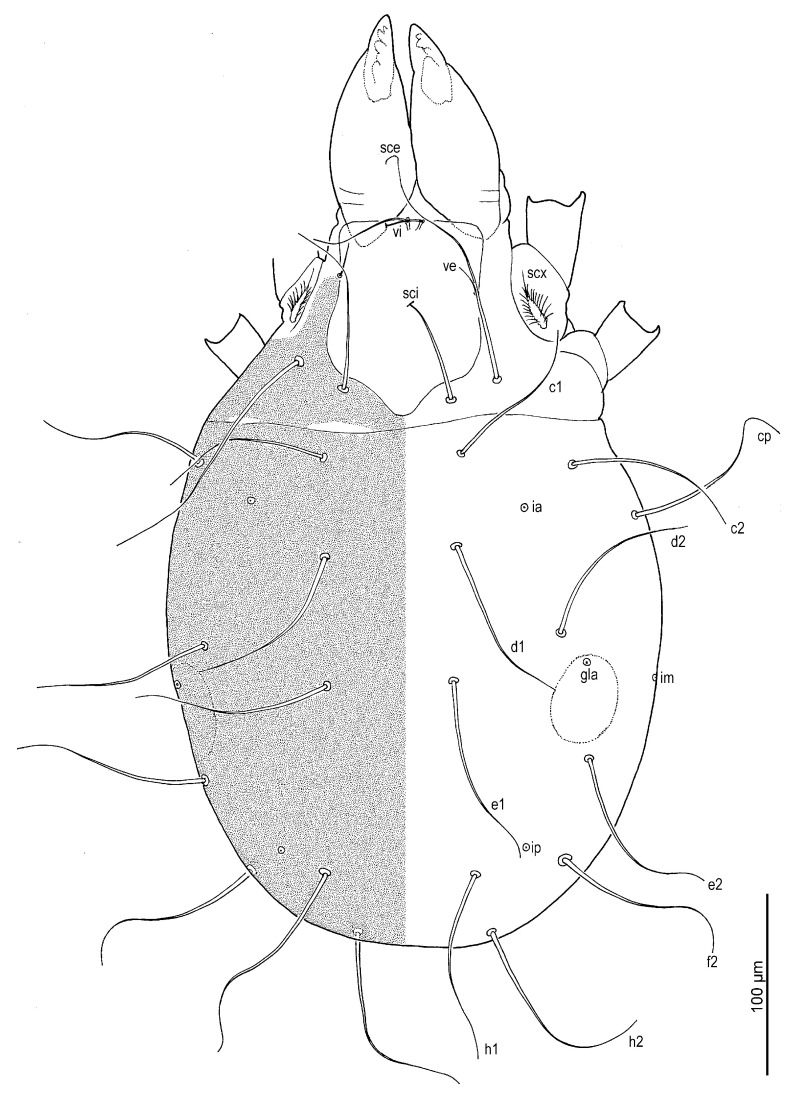
*Neosuidasia sjorsvandenbergi* sp. nov. (adult female). Idiosoma and chelicerae, dorsal view.

**Figure 2:**
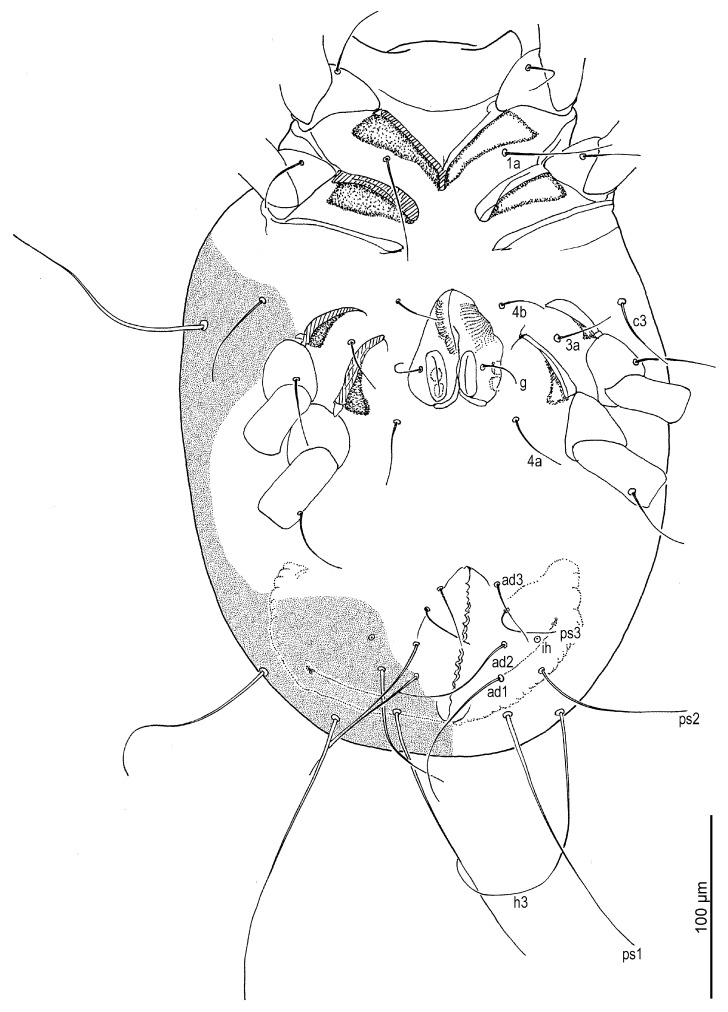
*Neosuidasia sjorsvandenbergi* sp. nov. (adult female). Idiosoma, ventral view.

**Figure 3:**
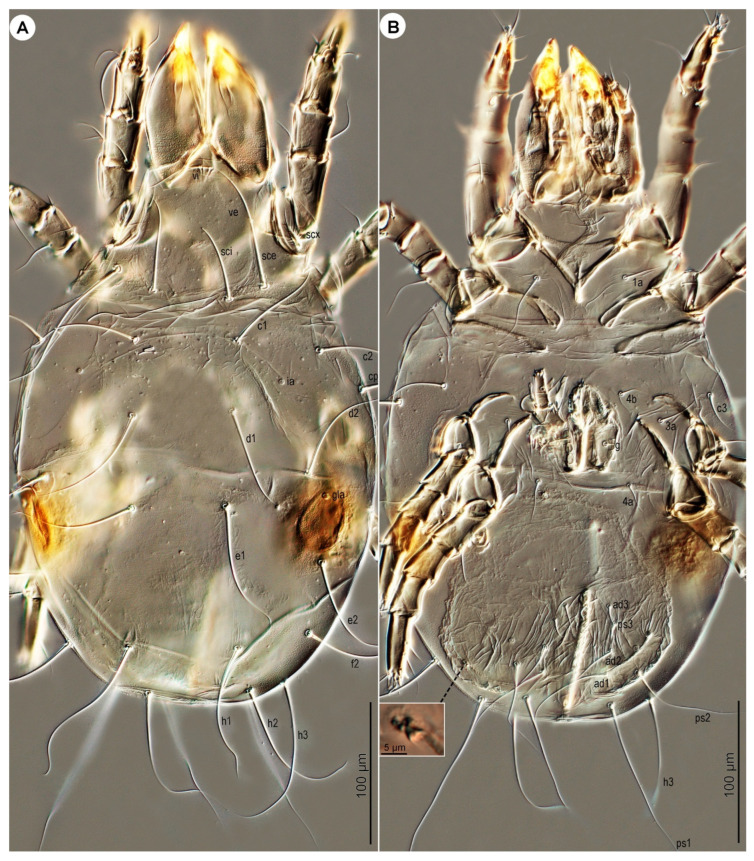
*Neosuidasia sjorsvandenbergi* sp. nov. (adult female, DIC images). (**A**). Dorsal view; (**B**). Ventral view (arrow: sclerite of the efferent duct).

**Redefinition.** ADULT FEMALE. Idiosomal cuticle (Figure 1) finely mamillated. Prodorsum with 4 pairs of setae (*vi*, *ve*, *sci*, and *sce*); hysterosoma with 12 pairs of setae (*c1*, *c2*, *cp*, *c3*, *d1*, *d2*, *e1*, *e2*, *f2*, *h1*, *h2*, and *h3*), all setae smooth and filiform. External vertical setae (*ve*), much shorter than internal vertical setae (*vi*), positioned at mediolateral margins of prodorsal shield, about anterior 1/3 of distance between *vi* and *sce*. Internal scapular setae (*sci*) shorter but exceeding half-length of external scapular setae (*sce*). Supracoxal setae (*scx*) basally expanded, gradually tapering distally, densely bearing long pectinations (Figure 3); Grandjean’s organ (Figure 3) a semi-circular plate, bordered by flame-like filaments. Most hysterosomal setae distinctly longer than distance to setae of next row. Genital opening (Figure 2) between coxae III–IV; genital and anal openings separated by distance exceeding genital opening length. Tibial setae conical and spine-like (Figure 6). Tarsal dorsal distal seta (*e*) slender spine, unguinal setae (*u* and *v*) setiform and small. Tarsus I solenidion *ω2* positioned slightly distal to *ω1*. Empodial claws well developed and condylophores narrower than empodial claw. Leg chaetotaxy (I–IV): coxae 1, 0, 1, 2; trochanters 1, 1, 1, 0; femora 1, 1, 0, 1; genua 2 + 2σ, 2 + 1σ, 1 + 1σ, 0; tibiae 2 + 1φ, 2 + 1φ, 1 + 1φ, 1φ; tarsi 7 attenuate setae + 1 slender spine + 2 minute setae + 3 ventral conical spines + 3ω + 1ε, 6 attenuate setae + 1 slender spine + 2 minute setae + 3 ventral conical spines + 1ω, 4 attenuate setae + 1 slender spine + 2 minute setae + 3 ventral conical spines, 4 attenuate setae + 1 slender spine + 2 minute setae + 3 ventral conical spines.

ADULT MALE. Similar to adult female (Figure 9). Genital opening approximately between trochanters IV, genital and anal openings close, separated by distance less than 1/3 length of genital opening. Adanal setae absent; para-anal suckers present. Two tarsal suckers (Figure 12) in distal half of tarsus IV. Posterior apodeme II wider, lateral part wider than medial part.

**Remarks.** *Neosuidasia* can be readily distinguished from other genera in Suidasiidae by its finely mamillated idiosomal cuticle, lacking protuberances (Figures 1 and 8) and spiniform tibial setae (Figures 6 and 12). Additional diagnostic features include: *ve* much shorter than *vi* and located at the mediolateral margins of the prodorsal shield (Figures 1 and 8); *sci* at least half the length of *sce* in adults (Figures 1 and 8); and well-developed empodial claws (Figures 6 and 12).

### 3.3. Neosuidasia sjorsvandenbergi *Fan & Faraji, sp. nov.*

(Figure 1, Figure 2, Figure 3, Figure 4, Figure 5, Figure 6, Figure 7, Figure 8, Figure 9, Figure 10, Figure 11, Figure 12, Figure 13, Figure 14, Figure 15, Figure 16, Figure 17, Figure 18, Figure 19, Figure 20, Figure 21, Figure 22, Figure 23, Figure 24, Figure 25, Figure 26 and Figure 27, Table 1)

urn:lsid:zoobank.org:pub:8DEEE2FB-FDAE-446E-B43E-8E23C3019F01

**Material examined**

Holotype female, paratypes 19 females, 12 males, 10 tritonymphs, 2 protonymphs, *ex* rearing culture, originally collected from a home poultry (open cage) in February 2022 in Monster, The Netherlands (52°02′18.6″ N, 4°12′27.4″ E), 20 January 2025, Sjors Van den Berg coll.; 26 females, 8 males, 5 tritonymphs, 2 protonymphs, 2 larvae, same collection data as the holotype but collected on 19 January 2024.

The holotype, 10 paratype females, 10 paratype males, 10 tritonymphs, 4 protonymphs and 2 larvae will be deposited in the New Zealand Arthropod Collection (NZAC), Landcare Research, Auckland. An additional 35 paratype females, 10 paratype males, and 5 tritonymphs are housed at the Plant Health and Environment Laboratory, Auckland, New Zealand (PANZ).

**Diagnosis**

This new species can be readily distinguished from the only previously known species, *N. faini* Ranganath & ChannaBasavanna, by the following characteristics: in adult females, setae *ps2* are 2.5 (1.7–2.5)× length of *ad3* (compared to slightly longer in *N. faini*) and about as long as anal opening (versus half its length in *N. faini*). In males, *ps2* is more than four times the length of *ps3*, while in *N. faini*, *ps2* is less than twice the length of *ps1*.

**Description**

**Figure 4:**
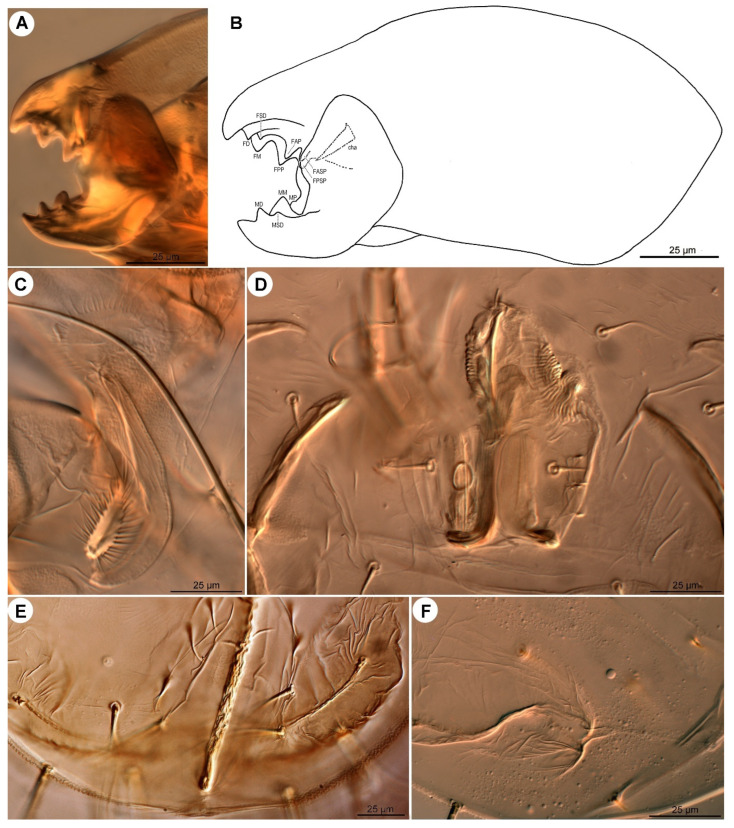
*Neosuidasia sjorsvandenbergi* sp. nov. (adult female). (**A**). Chelicera (DIC image), antiaxial view; (**B**). Chelicera (line drawing), antiaxial view; (**C**). Supracoxal sclerite, *scx* and Grandjean’s organ (DIC image); (**D**). Genital area (DIC image); (**E**). Receptaculum seminis and anal area (DIC image); (**F**). Inseminatory canal and receptaculum seminis (DIC image). **Abbreviations: *cha***, cheliceral seta; **FAP**, fixed digit, antiaxial proximal tooth; **FASP**, fixed digit, antiaxial subproximal tooth; **FD**, fixed digit, distal tooth; **FM**, fixed digit, medial tooth; **FPP**, fixed digit, paraxial proximal tooth; **FPSP**, fixed digit, paraxial subproximal tooth; **FSD**, fixed digit, subdistal tooth; MD, movable digit, distal tooth; MM, movable digit, medial tooth; MP, movable digit, proximal tooth; MSD, movable digit, subdistal tooth.

**Figure 5:**
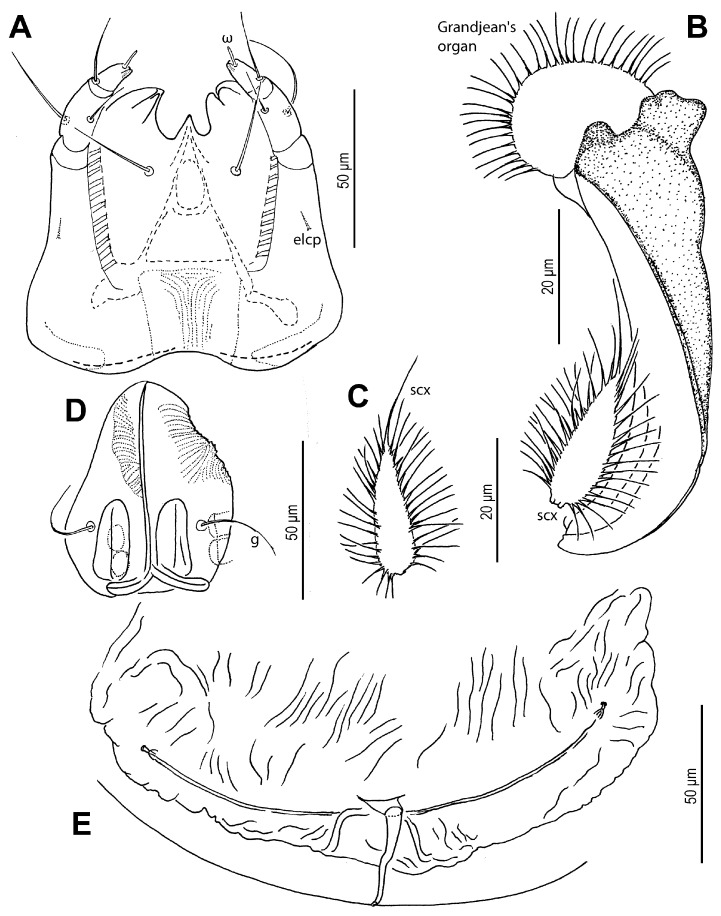
*Neosuidasia sjorsvandenbergi* sp. nov. (adult female). (**A**). Subcapitulum; (**B**). Supracoxal sclerite, *scx* and Grandjean’s organ; (**C**). *scx*; (**D**). Genital opening; (**E**), Inseminatory canal and receptaculum seminis.

**Figure 6:**
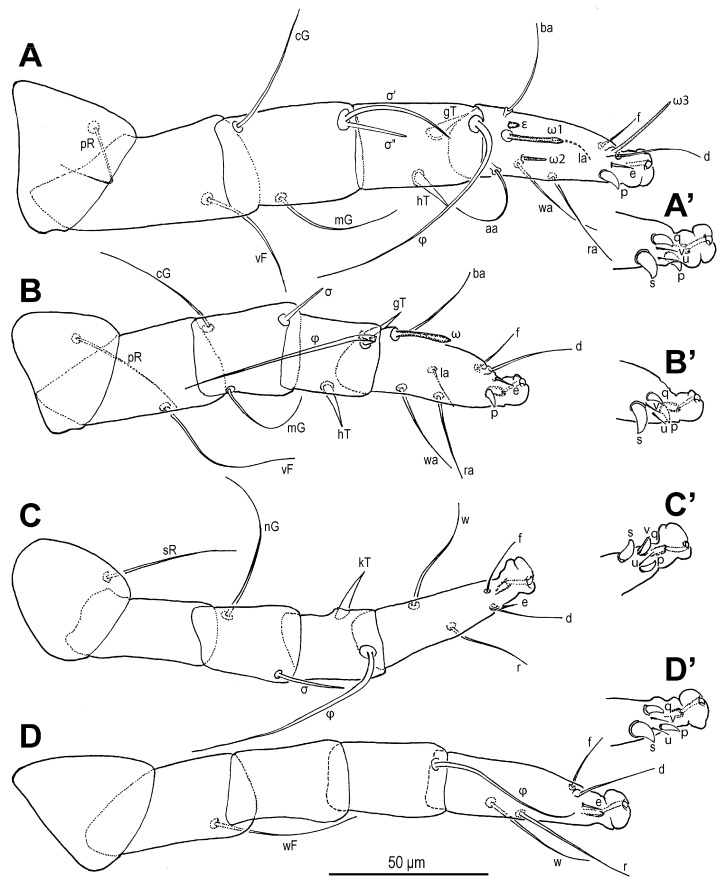
*Neosuidasia sjorsvandenbergi* sp. nov. (adult female). (**A**–**D**). Legs I–IV, dorsal view; (**A′**–**D′**). Pretarsi I–IV, ventral view.

**Figure 7:**
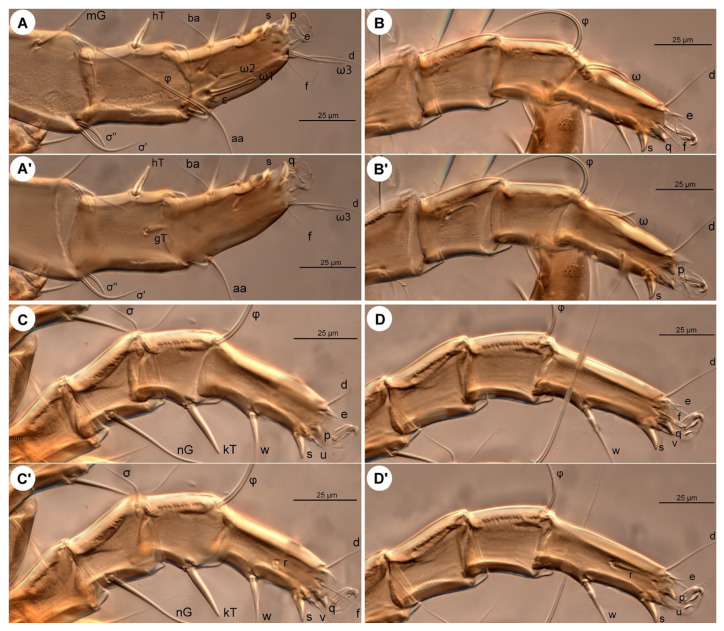
*Neosuidasia sjorsvandenbergi* sp. nov. (adult female, DIC images). (**A**–**D**). Genu–tarsus of legs I–IV, dorsal view; (**A′**–**D′**). Genu–tarsus of legs I–IV, ventral view.

ADULT FEMALE (Figure 1, Figure 2, Figure 3, Figure 4, Figure 5, Figure 6 and Figure 7; Table 1)

Dorsum (Figure 1 and Figure 3A) broadly pyriform; cuticle finely mammillated over almost entire dorsal surface, except for two narrow transverse areas anterior to sejugal furrow (Figure 1). Prodorsal shield anterior to scapular setae pentagonal; posteromedial margin convex and projecting beyond the level of setae *sci*; surface finely punctate. Supracoxal sclerite (Figure 4C and Figure 5B) elongate. Grandjean’s organ (Figure 4C and Figure 5B) semicircular in shape, with long marginal pectinations. Supracoxal setae *scx* (Figure 4C and Figure 5B,C) lanceolate, with 24–28 long marginal pectinations. Idiosomal setae filiform, *vi* slightly shorter than prodorsal shield width, *ve* much shorter than *vi*; located laterally, at anterior 1/4–1/3 of prodorsal shield; *sci* more than 4/5 length of *sce*; distance *sci*–*sci* 2.1 (1.8–2.1)× *sci*–*sce*. Opisthonotal gland openings *gla* positioned closer to *d2* than *e2*. Setae *c3* shortest, on ventral surface; remaining setae similar in length, extending beyond bases of next row setae. Cupules *ia* situated posterolateral to setae *c1* and posteromedial to *c2*; *im* located lateral to gland opening *gla*; *ip* positioned anterolateral to *h1* and anteromedial to *f2*.

Venter (Figure 2, Figure 3B, Figure 4D,F and Figure 5D,E): Cuticle finely mammillated in anterolateral region, lateral surfaces of coxae III–IV, and extending along lateral margin toward region near to anus. Coxal apodemes I fused medially, forming short prosternal apodeme directed posteromedially. Coxal plates I reach apex of prosternal apodeme; posterior margins slightly concave. Setae *1a* lateral to prosternal apodeme. Coxal apodemes II directed posteromedially; coxal plates II approximately twice as wide as apodemes II, extending to their apex; posterior margins nearly straight. Posterior apodemes of coxae II distinct, appearing as ridges, narrower than apodemes II. Genital opening (Figure 2, Figure 3B, Figure 4D and Figure 5D) inverted V-shaped, situated between coxae III and IV. Apodemes III and IV directed anteromedially. Setae *4b* lateral to anterior margin of genital opening; *g* lateral to first pair of genital papillae; *4a* posterolateral to lateral genital arms. Genital and anal openings separated by more than 1.3 (1.3–2.2)× genital opening length. Anal opening distinctly longer than genital opening; surrounded by 3 pairs of adanal setae (*ad1–3*) and 3 pairs of pseudanal setae (*ps1–3*). Setae *ad3* and *ps3* obviously shorter; *ps1* longest, 1.2 (1.1–1.3)× *ps2*, 2.8 (2.1–2.8)× *ps3*; *ps2* 2.5 (1.7–2.5)× length of *ad3* and nearly equal to anal opening length. Cupule *ih* situated lateral to setae *ad2*. Copulatory opening (Figure 4F and Figure 5E) near posterior margin of idiosoma. Inseminatory canal (spermathecal duct) (37 (36–48)) slender, cylindrical, slightly expanded at base of receptaculum seminis (spermathecal sac). Sclerites of efferent ducts (sclerites of oviducts) (4 (3–4)) bell-shaped, widely separated (112 (112–176)), approximately as wide as distance between trochanter IV bases.

Gnathosoma (Figure 4A,B and Figure 5A). Chelicerae robust, chelate-dentate (Figure 4A,B). Movable digit with 4 teeth; distal tooth (MD) nearly 3/4 of medial tooth (MM), subdistal (MSD) and proximal (MP) teeth indistinct, antiaxial and paraxial, respectively. Fixed digit with 7 teeth, 4 antiaxial and 3 paraxial, subdistal (FSD) obviously smaller than others; two most proximal teeth (FASP and FPSP) hard to observe. Cheliceral setae *cha* strong, conical. Subcapitulum (Figure 5A) with setae *h* filiform. Palpal supracoxal setae *elcp* minute and slender; palptibial setae (*sup*, *a*) and palptarsal seta (*cm*) filiform; terminal palptarsal solenidion (*ω*) distinct and finge-shaped; eupathidium *ul”* distally rounded.

Legs (Figure 6 and Figure 7). Legs I and IV distinctly longer than II and III. Setae (if present) on trochanters, femora, genua smooth, filiform; tibial setae (if present) conical, spiniform.

Leg I (Figure 6A,A′ and Figure 7A,A′): Genual solenidia ratio *σ′*:*σ″* = 1.6 (1.2–1.6). Tibial solenidion *φ* elongate, extending well beyond tarsal claw tip. Tarsus 2.3 (2.3–2.8)× as long as basal width. Solenidion *ω1* mostly cylindrical, tapering pre-apically into distinct terminal head; *ε* conical, near base of *ω1*; *ω2* cylindrical, slightly anterior to *ω1*; *ω3* distal, mostly cylindrical, apically tapered, as long as *ω1*. Seta *aa* filiform, proximal to *ω1*; *ba* filiform, level with *ω1*; *wa*, *ra*, and *la* filiform, *wa* and *ra* similar in length to *aa* and *ba*, and clearly longer than *la*; *d* filiform, distal, projecting well beyond claw tip; *e* a slender spine; *f* filiform, nearly reaching claw tip. Subunguinal seta (*s*) and proral setae (*p*, *q*) conical, ventrally curved; unguinal setae (*u*, *v*) slender, setiform.

Leg II (Figure 6B,B′ and Figure 7B,B′): Genual solenidion *σ* extending to half-length of tibia. Tibial solenidion *φ* elongate, extending beyond tarsal claw tip. Tarsus 2.1 (2.1–3.0)× as long as basal width. Solenidion *ω* mostly cylindrical, tapering pre-apically into distinct terminal head. Seta *bb* filiform, level with *ω*; *wa*, *ra*, and *la* filiform, *wa* and *ra* similar in length to *ba*, and clearly longer than *la*; *d* filiform, distal, projecting well beyond claw tip; *e* a slender spine; *f* filiform, about reaching claw tip. Subunguinal seta (*s*) and proral setae (*p*, *q*) conical, ventrally curved; unguinal setae (*u*, *v*) slender, setiform.

Leg III (Figure 6C,C′ and Figure 7C,C′): Genual solenidion *σ* extending over half-length of tibia. Tibial solenidion *φ* elongate, extending beyond tarsal claw tip. Tarsus 2.6 (2.6–3.1)× as long as basal width. Setae *w*, *r*, *d* and *f* filiform, *d* projecting well beyond claw tip; *e* a slender spine; *f* about reaching claw tip. Subunguinal seta (*s*) and proral setae (*p*, *q*) conical, ventrally curved; unguinal setae (*u*, *v*) slender, setiform.

Leg IV (Figure 6D,D′ and Figure 7D,D′): Tibial solenidion *φ* reaching tarsal claw tip or nearly so. Tarsus 2.7 (2.7–3.8)× as long as basal width. Setae *w*, *r*, *d*, and *f* filiform, *d* projecting well beyond claw tip; *e* a slender spine; *f* about reaching claw tip. Subunguinal seta (*s*) and proral setae (*p*, *q*) conical, ventrally curved; unguinal setae (*u*, *v*) slender, setiform.

**Table 1:** Measurements of morphological characters across life stages of *Neosuidasia sjorsvandenbergi* sp. nov.

	Females (*n* = 5)	Males (*n* = 5)	Tritonymphs (*n* = 5)	Protonymphs (*n* = 3)	Larvae (*n* = 2)
Idiosoma-L	398 (398–572)	353–417	281–366	208–271	216
Idiosoma-W	269 (269–389)	221–271	168–201	134–145	123
Shield-L	105 (103–136)	96–117	66–79	59–74	43
Shield-W	86 (82–115)	74–83	49–60	39–40	32–34
*vi*	63 (63–76)	61–62	31–35	18–24	20–29
*vi–vi*	7 (7–10)	6–9	5–6	5–6	4
*vi–ve*	50 (50–66)	41–50	28–34	20–28	22–23
*ve*	28 (27–36)	20–26	10–17	8–9	7–8
*ve–ve*	80 (80–108)	66–79	43–57	36–40	32–34
*sci*	99 (89–167)	103–119	44–57	18–25	12–16
*sci–sci*	58 (58–75)	50–64	36–41	34–36	15–28
*sci–sce*	28 (28–35)	28–31	20–28	14–18	15–17
*sce*	148 (148–197)	139–152	103–109	68–79	59
*scx*	41 (41–49)	29–34	18–26	17–21	12–15
*c1*	102 (102–143)	74–92	30–61	13–17	12–17
*c1–c1*	77 (77–112)	67–72	43–61	40–48	35–45
*c1–d1*	53 (46–68)	32–55	43–58	28–39	32
*c2*	109 (109–151)	78–112	47–67	24–36	14
*cp*	123 (123–169)	129–134	79–82	58–68	44
*c3*	62 (62–92)	52–63	27–35	20–23	14
*d1*	103 (103–154)	99–133	31–60	13–18	12
*d1–d1*	78 (76–107)	48–67	38–55	34–41	31
*d1–e1*	70 (57–117)	71–95	50–84	46–52	43
*d2*	101 (101–149)	89–112	44–59	14–22	12
*e1*	111 (111–152)	122–133	51–74	13–31	13
*e1–e1*	72 (72–105)	56–66	37–57	37–42	35
*e2*	116 (109–153)	118–121	58–84	39–58	32
*f2*	110 (110–145)	117–142	67–91	38–67	×
*h1*	125 (125–179)	119–129	68–71	37–63	48
*h2*	132 (132–158)	117–131	67–82	36–54	49
*h3*	153 (153–163)	136–156	87–119	87–109	×
*ps1*	117 (117–167)	102–134	69–102	31–49	×
*ps2*	97 (97–148)	41–56	39–48	17–26	×
*ps3*	42 (42–72)	11–13	14–20	9–13	×
*ad1*	85 (85–122)	×	×	×	×
*ad2*	77 (77–113)	×	×	×	×
*ad3*	39 (39–71)	×	×	×	×
Anus-L	90 (90–128)	66–74	53–64	39–47	36
*1a*	64 (59–77)	52–61	26–36	18–25	15
*4b*	34 (34–53)	28–35	15–19	×	×
*3a*	37 (37–71)	42–58	21–25	15–21	14
*g*	24 (24–33)	18–20	16–17	8–12	×
*4a*	41 (39–58)	31–49	×	×	×
Gen-L	67 (58–85)	44–50	24–29	14–16	×
Gen–anus	86 (86–144)	4–22	25–38	24–41	n/a
M-digit	46 (46–57)	42–48	27–30	17–23	16–17
*cha*	11 (10–15)	7–10	6–Jul	5–6	5–6
*h*	36 (34–44)	34–42	19–24	19–21	13–14
*elcp*	8 (8–10)	7–9	4–5	3–4	2
*sup*	27 (24–27)	28–37	15–20	13–15	12
*a*	24 (20–31)	20–36	13–14	10–12	9–10
*cm*	21 (21–27)	21–26	14–15	12–15	10–12
*ω*	8 (7–8)	7–9	4–5	4	3
Leg I	196 (196–289)	195–238	134–155	95–126	75–81
Fem-I-L	71 (70–89)	64–78	46–51	31–40	28–33
*vF I*	44 (44–54)	41–43	27–32	17–24	17–19
Genu-I-L	43 (41–59)	43–50	30–33	18–25	17
*cG I*	44 (44–77)	41–50	24–30	20–22	17–18
*mG I*	42 (42–61)	45–57	22–27	18–25	17
*σ′ I*	36 (36–47)	36–50	23–28	17–24	18–19
*σ″ I*	23 (23–32)	21–32	17–18	9–14	9
Ti-I-L	42 (41–55)	42–49	30–32	20–25	17–18
*gT I*	18 (17–30)	14–16	11–15	9–15	8–9
*hT I*	17 (17–32)	14–18	10–12	8–9	7
*φ I*	77 (77–92)	84–101	68–82	55–63	49
Ta-I-L	49 (49–72)	49–57	34–38	24–29	21–22
Ta-I-W	21 (21–26)	21–26	15–21	11–15	11–13
*ω1 I*	18 (18–22)	19–25	15–18	14–17	12–13
*ω2 I*	8 (8–12)	9–11	7–8	4–7	×
*ω3 I*	22 (22–25)	20–28	14–16	×	×
*ε I*	4 (3–4)	2–4	3–4	3	2
*aa*	31 (31–38)	29–36	16–21	15–19	9–10
*ba*	32 (30–37)	27–33	20–23	17–20	9–10
*wa I*	37 (33–41)	37–48	17–26	20–24	11–13
*la I*	22 (22–26)	19–25	11–16	11–15	7–9
*ra I*	31 (31–37)	30–43	15–20	17–20	12–13
*d I*	33 (31–36)	23–36	17–20	15–22	19–20
*e I*	6 (6–7)	7	5–6	4–5	3
*f I*	16 (16–18)	12–19	13–14	8–10	7–8
*s I*	12 (12–16)	9–12	8–9	5–7	4–5
*u I*	7 (6–7)	6–7	4–6	3–5	3–4
*v I*	7 (6–7)	6–7	4–6	3–5	3–4
*p I*	8 (8–10)	7–8	5–6	4–5	4–5
*q I*	8 (8–10)	7–8	5–6	4–5	4–5
Claw-I	10 (10–12)	8–11	7	6–7	5
Leg II	154 (154–233)	164–201	113–128	82–102	55–58
Fem-II-L	57 (57–75)	53–68	40–43	27–33	22–23
*vF II*	48 (44–65)	41–52	28–32	20–24	14–16
Genu-II-L	30 (30–45)	28–40	22–26	15–20	17–20
*cG II*	42 (42–66)	36–39	28–31	17–21	15–17
*mG II*	33 (31–65)	35–45	22–26	18–23	15–16
*σ II*	15 (15–19)	13–33	9–12	7–9	5–6
Ti-II-L	30 (30–40)	27–33	20–24	14–19	17–20
*gT II*	16 (16–26)	17–20	9–11	8–10	8
*hT II*	15 (15–19)	14–18	9–11	8–10	7
*φ II*	79 (79–98)	79–85	66–71	36–52	38–42
Ta-II-L	44 (44–64)	45–53	31–33	22–25	21–23
Ta-II-W	23 (18–23)	17–19	12–15	10–11	10–11
*ω II*	18 (18–22)	19–25	16–18	11–14	11–12
*ba II*	31 (31–36)	21–33	15–18	11–12	9–11
*wa II*	33 (33–42)	37–43	20–23	15–21	12–14
*la II*	20 (20–31)	16–20	12–14	9–13	7–8
*ra II*	33 (28–39)	27–37	17–22	15–20	12–13
*d II*	27 (24–29)	26–34	15–18	15–20	11–13
*e II*	6 (6–9)	6–7	5–7	4–5	3
*f II*	16 (15–17)	12–17	11–12	9–12	7–8
*s II*	12 (10–12)	7–9	7–8	5–7	4–5
*u II*	7 (6–7)	6	5–6	3–5	3–4
*v II*	7 (6–7)	6	5–6	3–5	3–4
*p II*	8 (7–8)	6–7	5–6	4–6	3–4
*q II*	8 (7–8)	6–7	5–6	4–6	3–4
Claw II	10 (10–11)	8–10	7	5–7	5
Leg III	152 (152–229)	162–188	115–121	69–94	79
Fem-III-L	47 (47–55)	44–52	32–34	21–28	21
Genu-III-L	30 (30–35)	29–34	21–23	15–19	16
*nG III*	46 (46–76)	49–65	34–36	17–23	18
*σ III*	21 (21–27)	14–25	14–16	9–13	7
Ti-III-L	28 (28–39)	29–31	21–22	15–19	18
*kT III*	16 (16–26)	19–23	13–15	10–13	12
*φ III*	79 (79–94)	74–86	45–62	44–54	42
Ta-III-L	46 (46–65)	43–50	27–32	18–28	42
Ta-III-W	18 (18–20)	15–27	12–13	9–10	11
*w III*	39 (39–50)	38–42	27–31	16–20	10
*r III*	27 (27–40)	36–39	14–19	11–15	8
*d III*	20 (20–29)	16–24	15–21	14–19	12
*e III*	7 (6–8)	7–8	5–6	5	4
*f III*	15 (15–17)	15–19	10–12	8–10	7
*s III*	9 (8–11)	8	5–6	4–5	5
*u III*	7 (7–8)	5–6	5–6	3	4
*v III*	7 (7–8)	5–6	5–6	3	4
*p III*	8 (7–8)	6–7	5–6	5	3
*q III*	8 (7–8)	6–7	5–6	5	3
Claw III	10 (10–11)	8–10	7	6–7	5
Leg IV	183 (183–263)	182–229	126–138	84–103	×
Fem-IV-L	51 (51–69)	41–58	34–36	20–26	×
*wF IV*	50 (50–67)	36–56	24–28	×	×
Gen-IV-L	34 (34–48)	31–41	22–24	15–19	×
Ti-IV-L	34 (33–45)	34–40	24–25	16–18	×
*φ IV*	54 (54–78)	46–66	28–35	×	×
Ta-IV-L	51 (51–75)	45–62	35–38	23–28	×
Ta-IV-W	19 (19–22)	17–22	11–12	9–10	×
*w IV*	35 (35–51)	37–51	27–30	16–21	×
*r IV*	31 (31–45)	34–45	17–24	13–17	×
*d IV*	31 (25–33)	●	19–22	14–20	×
*e IV*	7 (7–8)	●	4–6	×	×
*f IV*	16 (16–20)	16–22	10–11	×	×
*s IV*	9 (9–12)	8–10	6–7	■	×
*u IV*	6 (6–9)	5–8	5–6	3	×
*v IV*	6 (6–9)	5–8	5–6	5	×
*p IV*	7 (7–8)	6–8	6	4–6	×
*q IV*	7 (7–8)	6–8	6	4–6	×
Claw IV	10 (10–11)	8–10	7–8	6–7	×

×: absent; ■: rudimentary; ●: sucker-like.

**Figure 8:**
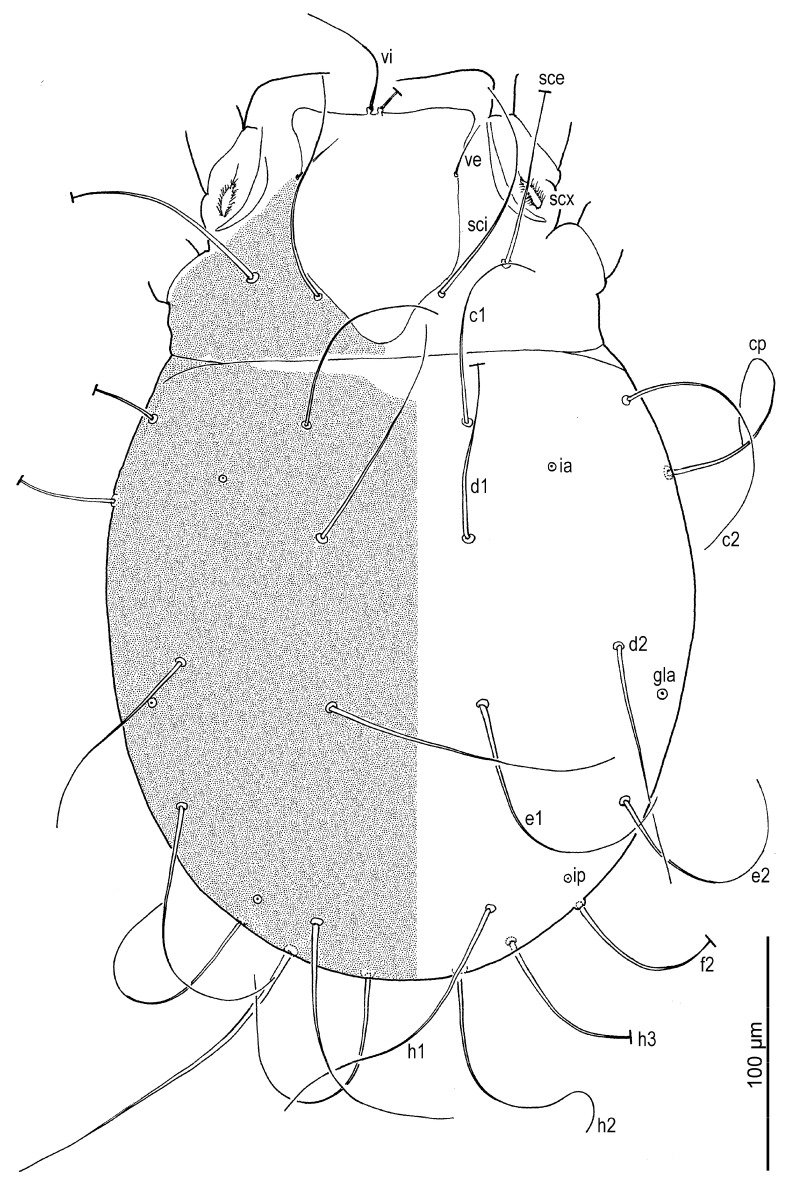
*Neosuidasia sjorsvandenbergi* sp. nov. (adult male). Idiosoma, dorsal view.

**Figure 9:**
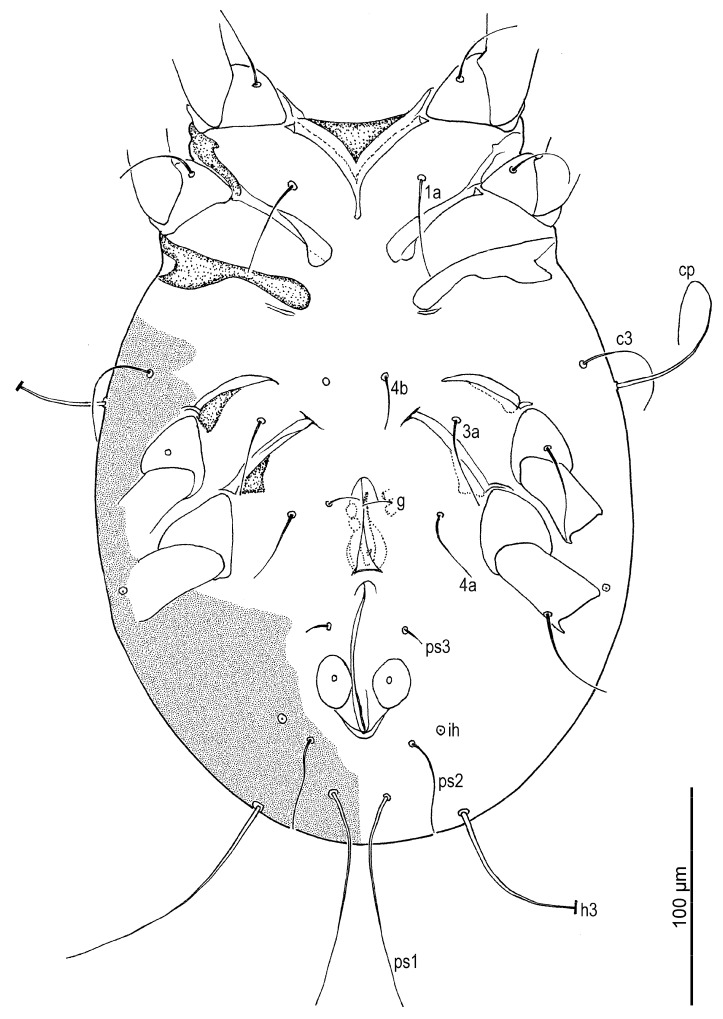
*Neosuidasia sjorsvandenbergi* sp. nov. (adult male). Idiosoma, ventral view.

**Figure 10:**
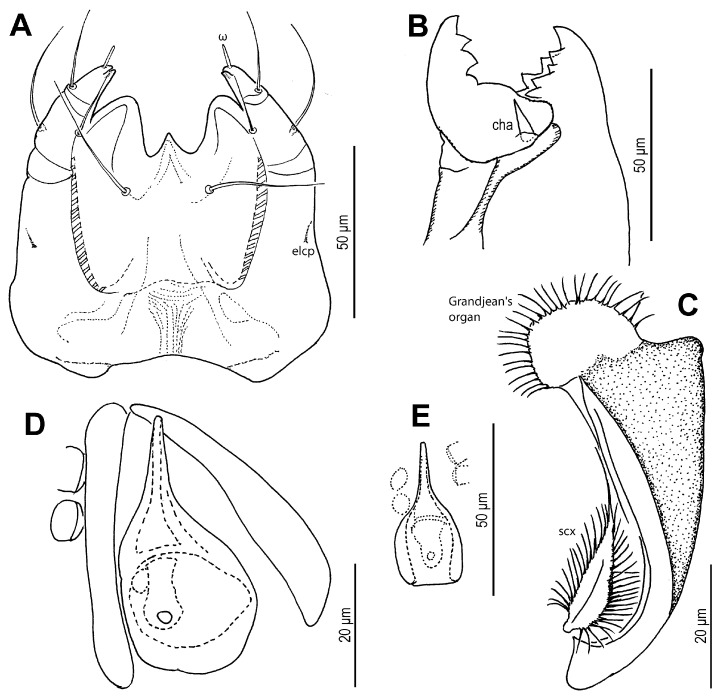
*Neosuidasia sjorsvandenbergi* sp. nov. (adult male). (**A**). Subcapitulum; (**B**). Chelicera; (**C**). Supracoxal sclerite, *scx,* and Grandjean’s organ; (**D**). Genital opening and aedeagus; (**E**). Aedeagus.

**Figure 11:**
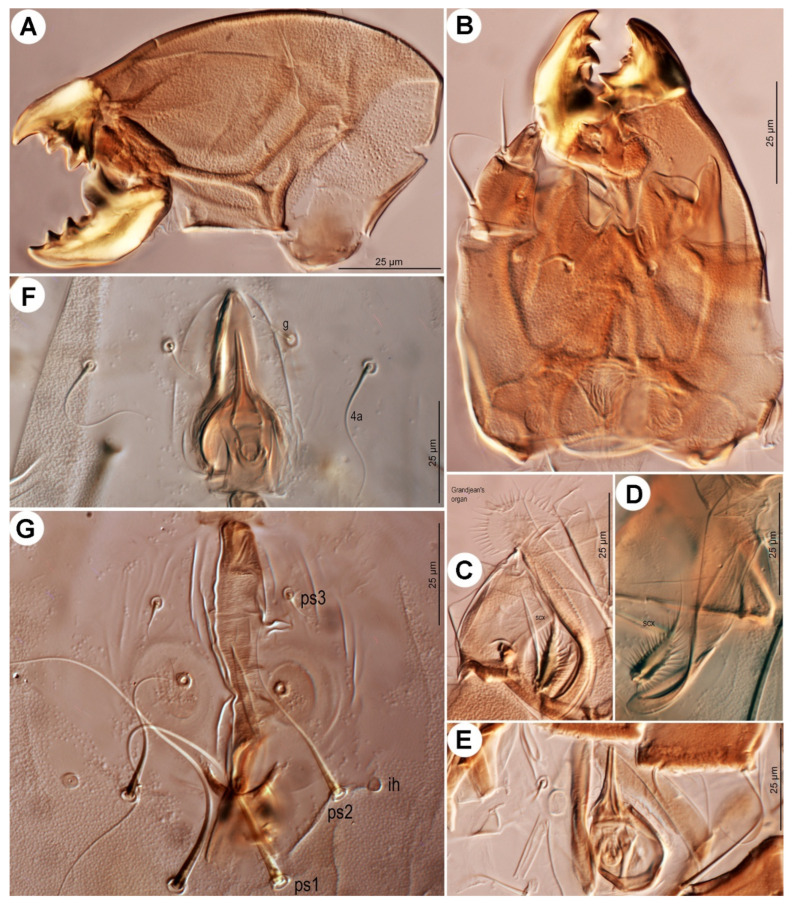
*Neosuidasia sjorsvandenbergi* sp. nov. (adult male, DIC images). (**A**). Chelicera; (**B**). Subcapitulum and chelicera; (**C**,**D**). Supracoxal sclerite, *scx,* and Grandjean’s organ; (**E**,**F**). Genital opening and aedeagus; (**G**). Anal area.

**Figure 12:**
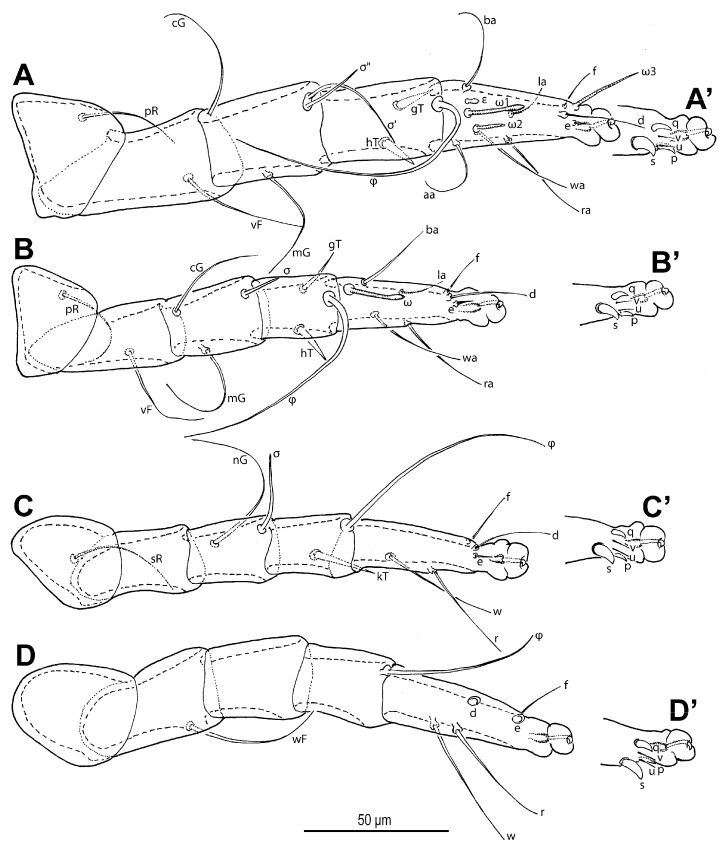
*Neosuidasia sjorsvandenbergi* sp. nov. (adult male). (**A**–**D**). Legs I–IV, dorsal view; (**A′**–**D′**). Pretarsi I–IV, ventral view.

**Figure 13:**
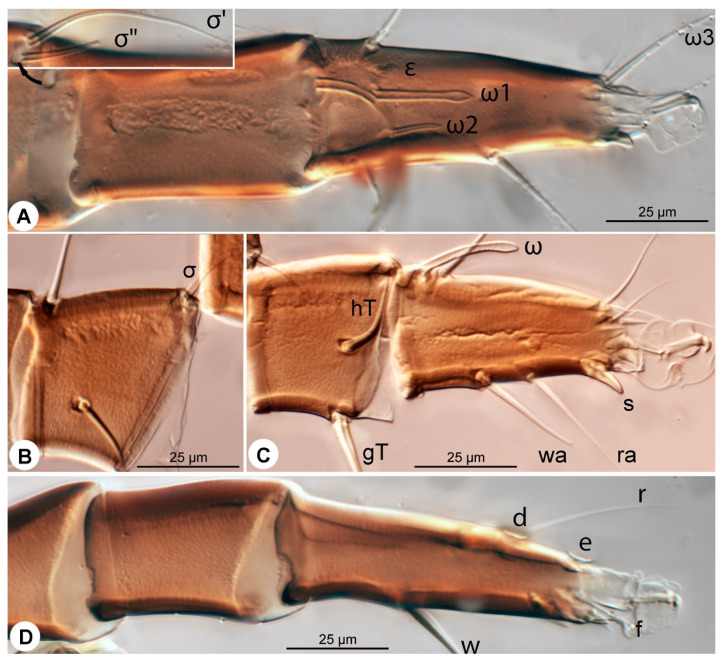
*Neosuidasia sjorsvandenbergi* sp. nov. (adult male, DIC images). (**A**). Genu–tarsus of legs I, dorsal view; (**B**). Genu of legs II, dorsal view; (**C**). Tibia and tarsus of legs II, dorsal view; (**D**). Genu–tarsus of leg IV, dorsal view.

Male (Figure 8, Figure 9, Figure 10, Figure 11, Figure 12 and Figure 13, Table 1)

Dorsum (Figure 8, Figure 10C and Figure 11C,D) broadly pyriform to oval; cuticle finely mammillated over nearly entire dorsal surface except anteromedial region between sejugal suture and level of *c1*. Prodorsal shield, supracoxal sclerite, Grandjean’s organ, and supracoxal setae (*scx*) as in adult female. Idiosomal setae filiform; *vi* ~3/4 prodorsal shield width; *ve* slightly longer than 1/3 *vi*; *sci* 4/5 length of *sce*; distance *sci–sci* 1.7–2.0× *sci–sce.* Distance *gla–e2* ~2× *gla–d2*. Setae *c3* shortest, positioned ventrally; remaining setae subequal, long, extending beyond bases of following row. Cupules: *ia* posterolateral to *c1* and posteromedial to *c2*; *im* on ventral surface, lateral to coxa IV; *ip* anteromedial to and near *f2*.

Venter (Figure 9, Figure 10D,E and Figure 11E–G): Cuticle finely mammillated across anterolateral regions of coxae III, lateral surfaces of coxae III–IV, posterior to coxae IV, and along lateral areas near anus. Coxal apodemes and plates as in adult female, except posterior apodemes of coxae II broader, lateral portion approximately 2× maximum width of coxa II. Positions of coxisternal setae *1a*, *4b*, and *4a* as in adult female. Genital opening (Figure 9, Figure 10D and Figure 11E,F) inverted V-shaped, markedly narrower than in female, located between trochanters IV; setae *g* at level of first pair of genital papillae. Genital and anal openings close, separated by less than half length of genital opening. Aedeagus (Figure 10D,E and Figure 11E,F) straight, gradually tapered, 44–46 long, extending nearly to anterior edge of supporting sclerites. Anal opening approximately 2× genital opening length. Adanal setae absent. Anal suckers pair, faintly sclerotised, posterolateral to anal opening. Pseudanal setae: *ps1* posterior to anus, *ps2* posterolateral to anus, *ps3* anterior to anal suckers; *ps1* longest, 4.6–4.7× *ps2*, 10.0–11.0× *ps3*; *ps2* 4.2–4.7× *ps3*. Cupule *ih* anterolateral to *ps2*.

Gnathosoma (Figure 10A,B and Figure 11A,B). Chelicerae teeth, seta *cha*, subcapitular seta *h*, palpal supracoxal seta *elcp*, palptibial setae *sup* and *a*, palptarsal seta *cm*, palptarsal solenidion *ω*, eupathidium *ul″* as in adult female, except for differences in dimensions.

Legs (Figure 12 and Figure 13): Legs I and IV longer and wider than II and III. Setal formulae identical to female, except tarsus IV with two suckers, modified from setae *d* and *e*.

Leg I (Figure 12A,A′ and Figure 13A): Genual solenidia ratio *σ′*:*σ″* = 1.5–1.9. Tibial solenidion *φ* extending far beyond tarsal claw tip. Tarsus 2.2–2.6× basal width. Solenidion *ω1* cylindrical with terminal head; *ε* apically tapered, near base of *ω1*; *ω2* cylindrical, slightly anterior to *ω1*; *ω3* dorsal distal, tapered, longer than *ω1*. Setae *aa*, *ba*, *wa*, *ra*, *la*, *d*, *f* filiform; *e* slender spine. Subunguinal seta (*s*), proral setae (*p*, *q*), and unguinal setae (*u*, *v*) as in adult female.

Leg II (Figure 12B,B′ and Figure 13B,C): Genual solenidion *σ* extending near half-length of tibia; *φ* extending far beyond tarsal claw tip. Tarsus 2.7–2.8× basal width. Solenidion *ω* cylindrical with terminal head. Setae *ba*, *wa*, *ra*, *la*, *d*, *f* filiform; e slender spine. Subunguinal seta (*s*), proral setae (*p*, *q*), and unguinal setae (*u*, *v*) as in adult female.

Leg III (Figure 12C,C′): Genual solenidion *σ* reaching base of tibial solenidion *φ*; *φ* extending far beyond tarsal claw tip. Tarsus 1.7–2.3× basal width. Setae *w*, *r*, *d*, *f* filiform; *e* slender spine. Subunguinal seta (*s*), proral setae (*p*, *q*), and unguinal setae (*u*, *v*) as in adult female.

Leg IV (Figure 12D,D′ and Figure 13D): Tibial solenidion *φ* slightly passing *f*. Tarsus 2.9–3.3× basal width. Setae *w*, *r*, *f* filiform; *d* and *e* sucker-like, slightly wider than alveolus of *w* or *r*, distance *d*–*e* 11–17 Subunguinal seta (*s*), proral setae (*p*, *q*), and unguinal setae (*u*, *v*) as in adult female.

**Figure 14:**
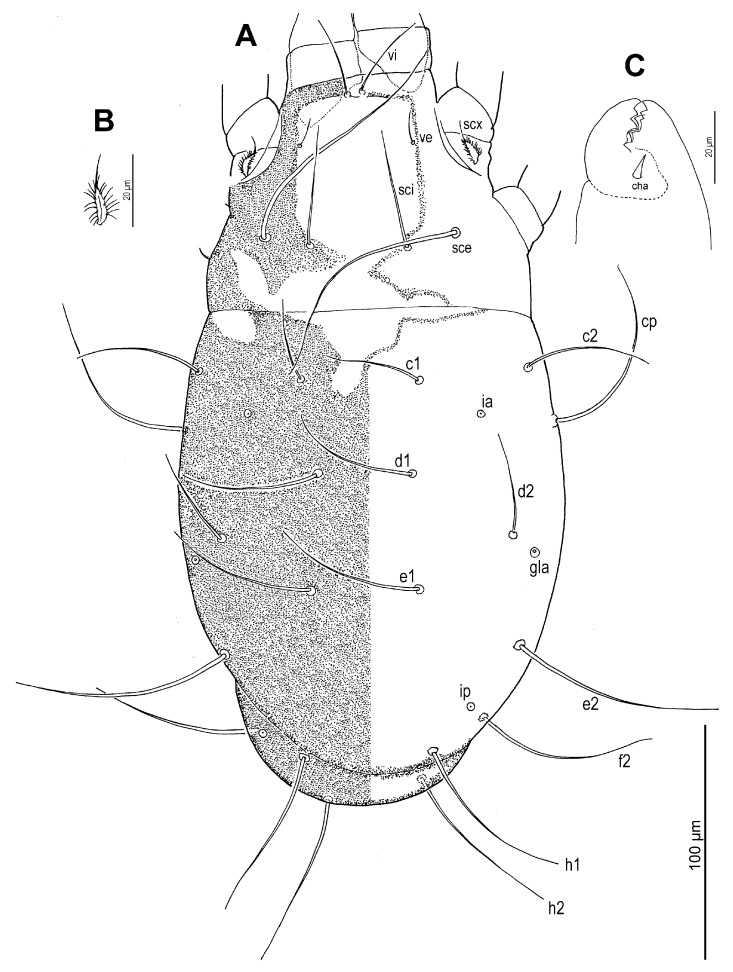
*Neosuidasia sjorsvandenbergi* sp. nov. (tritonymph). (**A**). Idiosoma, dorsal view; (**B**). *scx*; (**C**). Chelicera.

**Figure 15:**
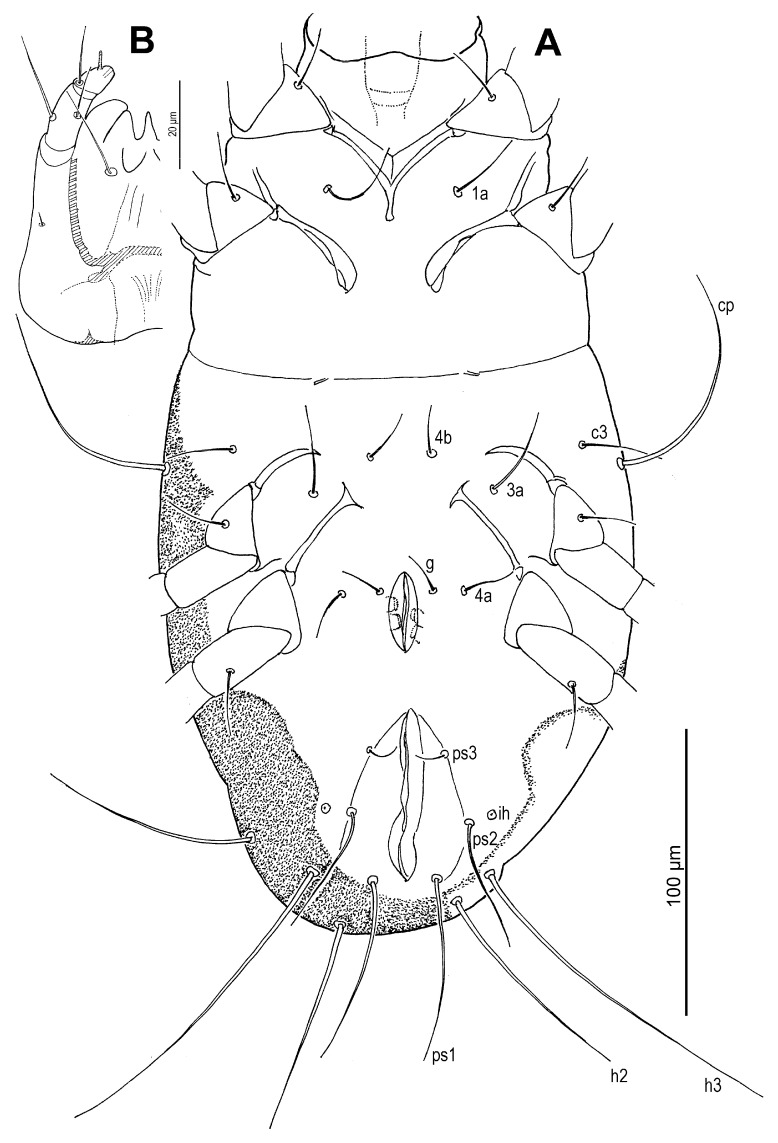
*Neosuidasia sjorsvandenbergi* sp. nov. (tritonymph). (**A**). Idiosoma, ventral view; (**B**). Subcapitulum.

**Figure 16:**
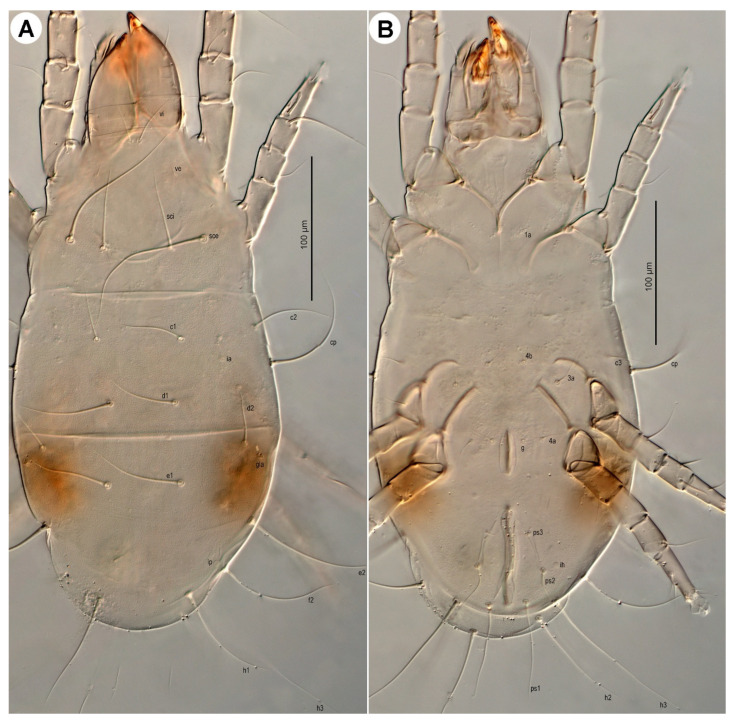
*Neosuidasia sjorsvandenbergi* sp. nov. (tritonymph, DIC images). (**A**). Dorsal view; (**B**). Ventral view.

**Figure 17:**
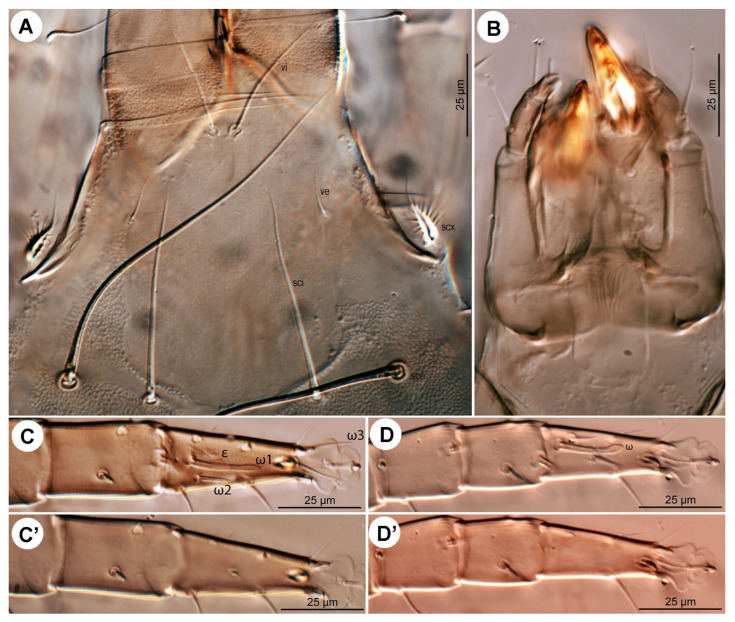
*Neosuidasia sjorsvandenbergi* sp. nov. (tritonymph, DIC images). (**A**). Prodorsum; (**B**). Subcapitulum; (**C**). Tibia and tarsus of legs I, dorsal view; (**C′**). Tibia and tarsus of legs I, ventral view; (**D**). Tibia and tarsus of legs II, dorsal view; (**D′**). Tibia and tarsus of legs II, ventral view.

**Figure 18:**
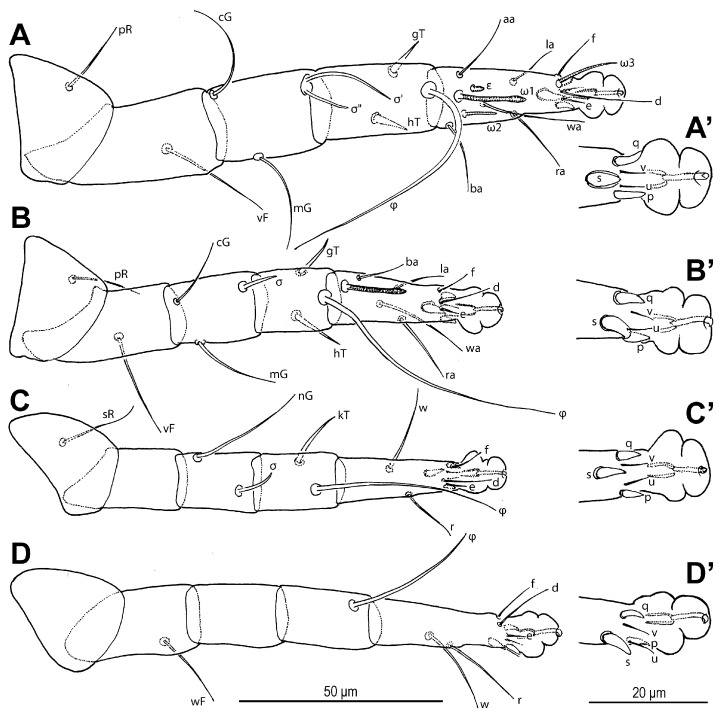
*Neosuidasia sjorsvandenbergi* sp. nov. (tritonymph). (**A**–**D**). Legs I–IV, dorsal view; (**A′**–**D′**). Pretarsi I–IV, ventral view.

Tritonymph (Figure 14, Figure 15, Figure 16, Figure 17 and Figure 18, Table 1)

Dorsum (Figure 14A,B, Figure 16A and Figure 17A) elongated oval; cuticle finely mammillated across most of dorsal surface, except near sejugal suture, specifically posterior to scapular setae and anteromedial and medial to *c1*. Prodorsal shield rectangular anterior to *sci*, narrowing posteromedially; supracoxal sclerite and Grandjean’s organ as in adult female; *scx* pectinate, bearing 20–26 long teeth. Idiosomal setae filiform; *vi* about 1/2 prodorsal shield width; *ve* less than 1/2 *vi*; *sci* 1/2 length of *sce* or longer; distance *sci*–*sci* 1.5–1.6× *sci*–*sce*. Distance *gla*–*e2* near 3× *gla*–*d2*. Setae *c3* shortest, lateral to coxae III; others extending beyond or near bases of following row. Cupules: *ia* posteromedial to *c2*; *im* marginal; *ip* close to and anteromedial to *f2*.

Venter (Figure 15A and Figure 16B): Cuticle finely mammillated across regions lateral to coxae III–IV, extending posterior beyond coxae IV and laterally to level of cupules *ih*. Coxal apodemes and plates similar to those of female, except posterior apodemes of coxae II indistinct. Coxisternal setae *1a*, *4b*, and *4a* positioned as in adult female. Genital opening a longitudinal slit between trochanters IV; setae *g* at level of *4a*. Distance between genital and anal openings near length of genital slit. Anal opening more than twice length of genital opening. Adanal setae absent. Pseudanal setae *ps1* at same level of posterior margin of anus; *ps2* and *ps3* located laterally; *ps1* longest, 1.6–2.0× *ps2*, 3.8–6.4× *ps3*; *ps2* 2.3–3.3× *ps3*. Cupule *ih* anterolateral to *ps2*.

Gnathosoma (Figure 14C, Figure 15B, Figure 16B and Figure 17B): Structure and setation of chelicerae, subcapitulum, and palps as in adult female, except for differences in dimensions.

Legs (Figure 17 and Figure 18): Legs I and IV longer than legs II and III. Setal formulae same as those of female.

Leg I (Figure 17C,C′ and Figure 18A,A′): Ratio *σ′*:*σ″* = 1.3–1.7. Tibial solenidion φ extends well beyond tarsal claw tip. Tarsus length 1.7–2.3× basal width. Solenidion *ω1* cylindrical with terminal head; *ε* tapered apically, located anterior to *ω1* base; *ω2* cylindrical, slightly anterior to *ω1*; *ω3* positioned dorsodistally, tapered, slightly shorter than *ω1*. Setae *aa*, *ba*, *wa*, *ra*, *la*, *d*, and *f* filiform; *e* setiform. Subunguinal seta (*s*), proral setae (*p*, *q*), and unguinal setae (*u*, *v*) as in adult female.

Leg II (Figure 17D,D′ and Figure 18B,B′): Genual solenidion *σ* extending to nearly one-third of tibial length; tibial solenidion *φ* extends well beyond tarsal claw tip. Tarsus 2.1–2.4× basal width. Solenidion *ω* cylindrical with terminal head. Setae *ba*, *wa*, *ra*, *la*, *d*, *f* filiform; *e* setiform. Subunguinal seta (*s*), proral setae (*p*, *q*), and unguinal setae (*u*, *v*) as in adult female.

Leg III (Figure 18C,C′): Genual solenidion *σ* reaching about one-third of tibial length; tibial solenidion *φ* reaches tarsal claw tip. Tarsus 2.4–2.7× basal width. Setae *w*, *r*, *d*, *f* filiform; *e* setiform. Subunguinal seta (*s*), proral setae (*p*, *q*), and unguinal setae (*u*, *v*) as in adult female.

Leg IV (Figure 18D,D′): Tibial solenidion *φ* nearly reaching base of seta *f*. Tarsus length 3.0–3.2× basal width. Setae *w*, *r*, *d*, *f* filiform; *e* setiform. Subunguinal seta (*s*), proral setae (*p*, *q*), and unguinal setae (*u*, *v*) as in adult female.

**Figure 19:**
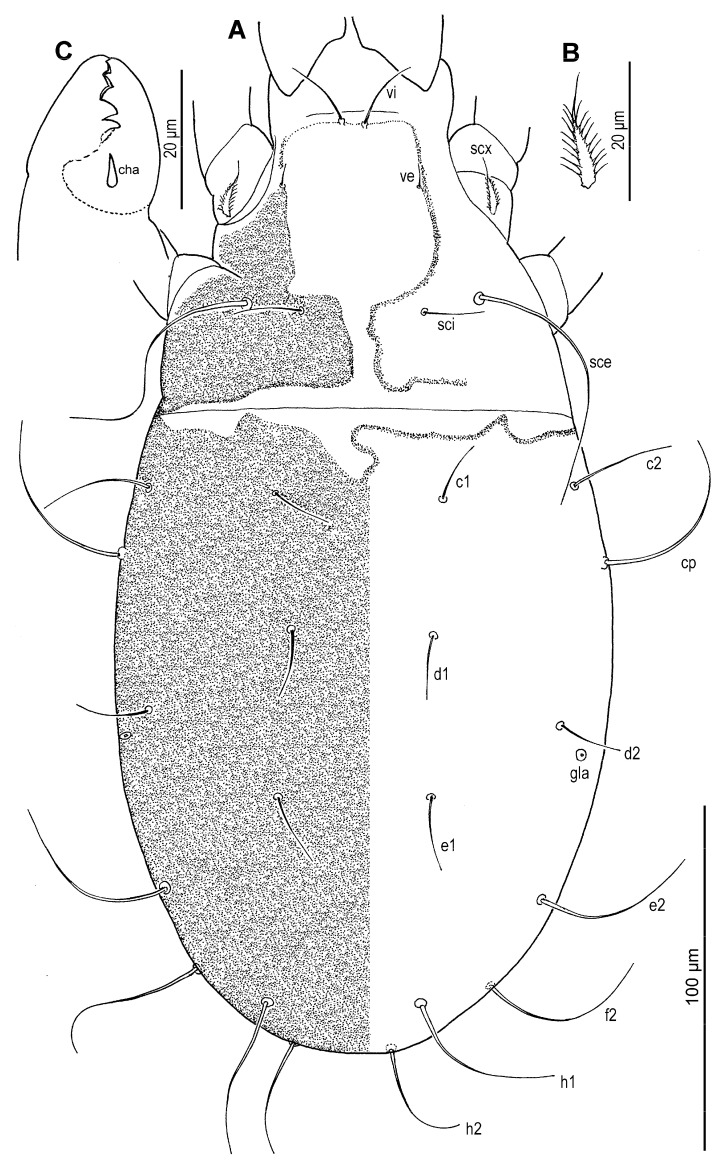
*Neosuidasia sjorsvandenbergi* sp. nov. (protonymph). (**A**). Idiosoma, dorsal view; (**B**). *scx*; (**C**). Chelicera.

**Figure 20:**
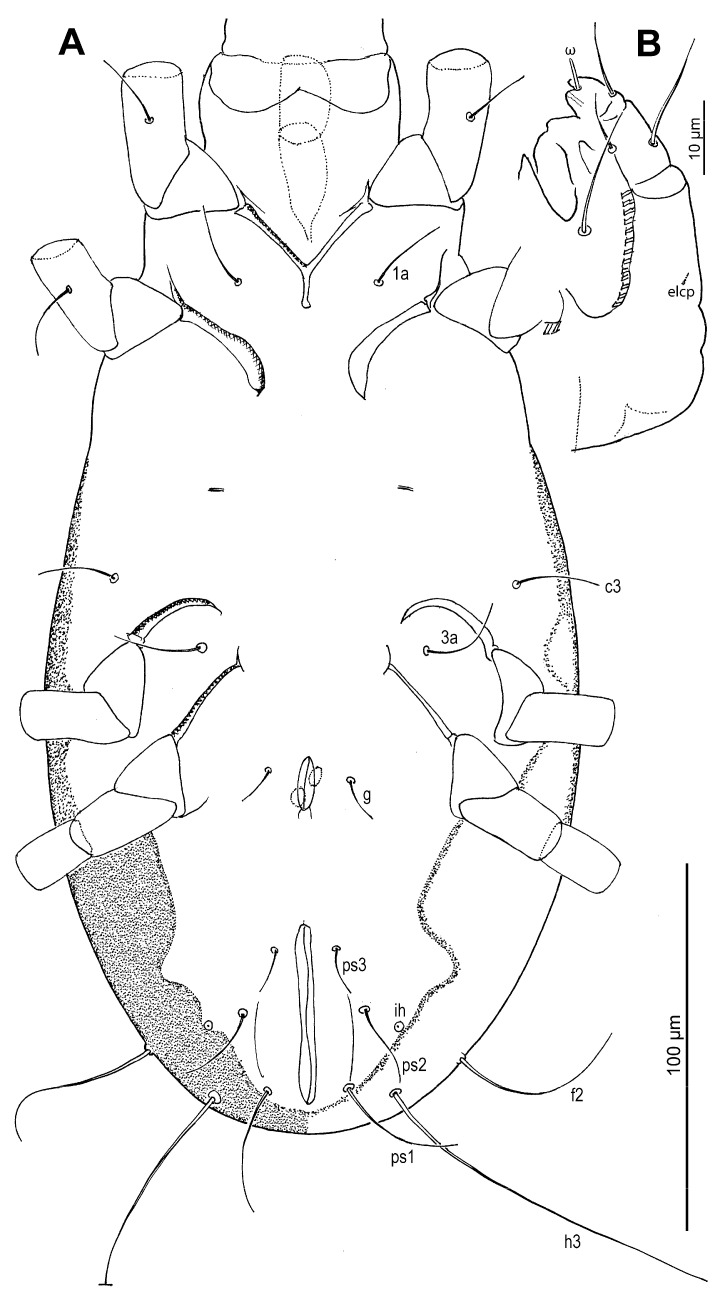
*Neosuidasia sjorsvandenbergi* sp. nov. (protonymph). (**A**). Idiosoma, ventral view; (**B**). Subcapitulum.

**Figure 21:**
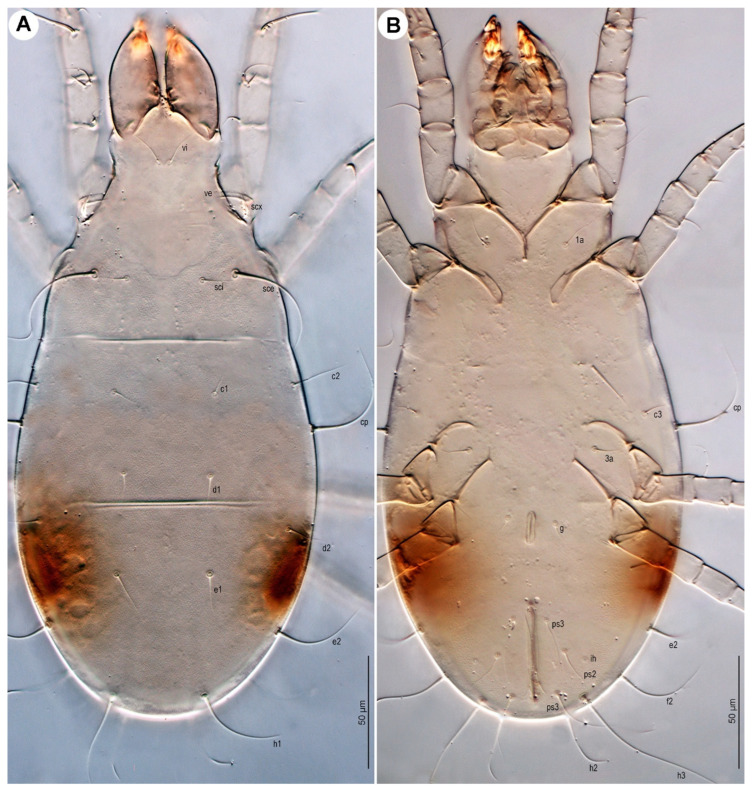
*Neosuidasia sjorsvandenbergi* sp. nov. (protonymph, DIC images). (**A**). Dorsal view; (**B**). Ventral view.

**Figure 22:**
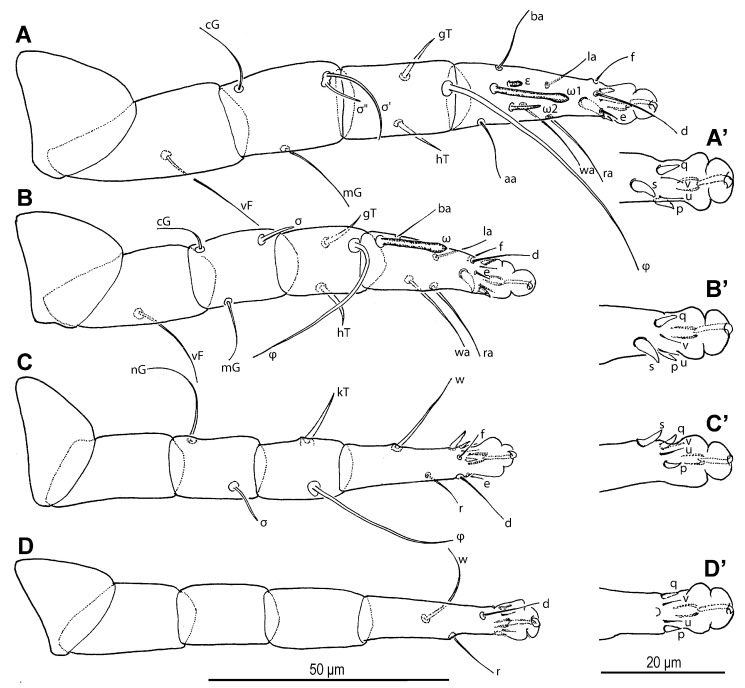
*Neosuidasia sjorsvandenbergi* sp. nov. (protonymph). (**A**–**D**). Legs I–IV, dorsal view; (**A′**–**D′**). Pretarsi I–IV, ventral view.

Protonymph (Figure 19, Figure 20, Figure 21 and Figure 22, Table 1)

Dorsum (Figure 19A,B and Figure 21A) elongated oval; cuticle finely mammillated across most of dorsal surface, except along sejugal suture. Prodorsal shield rectangular, with posteromedial extension. Supracoxal sclerite and Grandjean’s organ as in adult female; *scx* pectinate with 18–24 teeth. Idiosomal setae filiform; *vi* approximately 0.7× width of prodorsal shield; *ve* about one-quarter length of *vi*. Setae *sci* less than one-third length of *sce*; distance *sci*–*sci* 2.0–2.1× *sci*–*sce*. Distance *gla*–*e2* nearly 4× *gla*–d2. Setae *c3* situated anterolateral to coxae III, subequal to *c1*, *d1*, and *d2*. Setae *c1*, *d1*, *d2*, and *e2* well separated from bases of following row. Cupules indistinct.

Venter (Figure 20A and Figure 21B): Mammillated area reduced compared to tritonymph, restricted to lateral margins lateral to coxae III–IV and anal region. Coxal apodemes and plates similar to female, without indistinct posterior apodemes of coxae II. Coxisternal setae *4a* and *4b* absent. Genital opening a short longitudinal slit between trochanters IV; setae *g* lateral to anterior part of Genital opening. Distance between genital and anal openings approximately 2× length of genital slit. Anal opening nearly 3× longer than genital slit. Adanal setae absent. Pseudanal setae: *ps1* at level of posterior anus margin; *ps2* and *ps3* lateral. Seta *ps1* longest, 1.4–1.6× *ps2*, 3.8–4.0× *ps3*; *ps2* 2.5–2.8× *ps3*. Cupule *ih* positioned posterolaterally to *ps2*.

Gnathosoma (Figure 19C, Figure 20B and Figure 21B): Chelicerae, subcapitulum, and palpal setation as in adult female, differing only in dimensions.

Legs (Figure 22): Leg I distinctly longer than others. Trochanters, femur IV, and tibia IV nude.

Leg I (Figure 22A,A′): Genual solenidia ratio *σ′:σ″* = 1.6–1.7. Tibial solenidion *φ* extends well beyond tarsal claw tip. Tarsus 1.8–2.0× basal width. Solenidion *ω1* cylindrical with terminal head; *ε* apically tapered, positioned anterolateral to base of *ω1*; *ω2* cylindrical, slightly anterolateral to *ω1*; *ω3* absent. Setae *aa*, *ba*, *wa*, *ra*, *la*, *d*, *f* filiform; *e* setiform. Subunguinal seta (*s*), proral (*p*, *q*), and unguinal setae (*u*, *v*) as in adult female.

Leg II (Figure 22B,B′): Genual solenidion *σ* reaching nearly one-fourth of tibial length; tibial solenidion *φ* extends to tarsal claw tip. Tarsus 2.1–2.2× basal width. Solenidion *ω* cylindrical with terminal head. Setae *ba*, *wa*, *ra*, *la*, *d*, *f* filiform; *e* setiform. Subunguinal seta (*s*), proral (*p*, *q*), and unguinal setae (*u*, *v*) as in adult female.

Leg III (Figure 22C,C′): Genual solenidion σ reaches about one-fourth of tibial length; *φ* nearly reaches base of seta *d*. Tarsus 2.7–2.8× basal width. Setae *w*, *r*, *d*, *f* filiform; *e* setiform. Subunguinal seta (*s*), proral (*p*, *q*), and unguinal setae (*u*, *v*) as in adult female.

Leg IV (Figure 22D,D′): Tibial solenidion *φ* absent. Tarsus 3.7–3.8× basal width. Setae *e* and *f* absent; *w*, *r*, *d* filiform. Subunguinal seta (*s*) undeveloped; proral (*p*, *q*), and unguinal setae (*u*, *v*) as in adult female.

**Figure 23:**
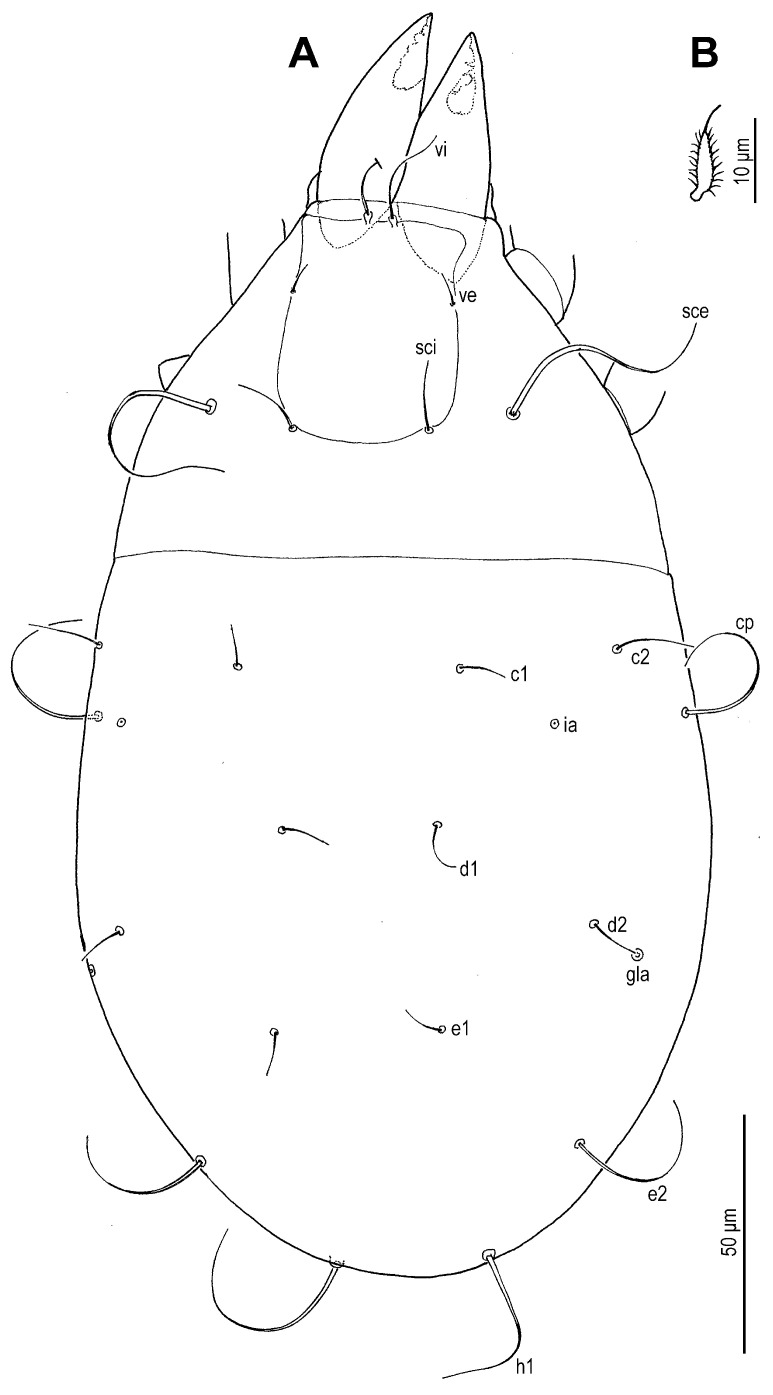
*Neosuidasia sjorsvandenbergi* sp. nov. (larva). (**A**). Idiosoma, dorsal view; (**B**). *scx*.

**Figure 24:**
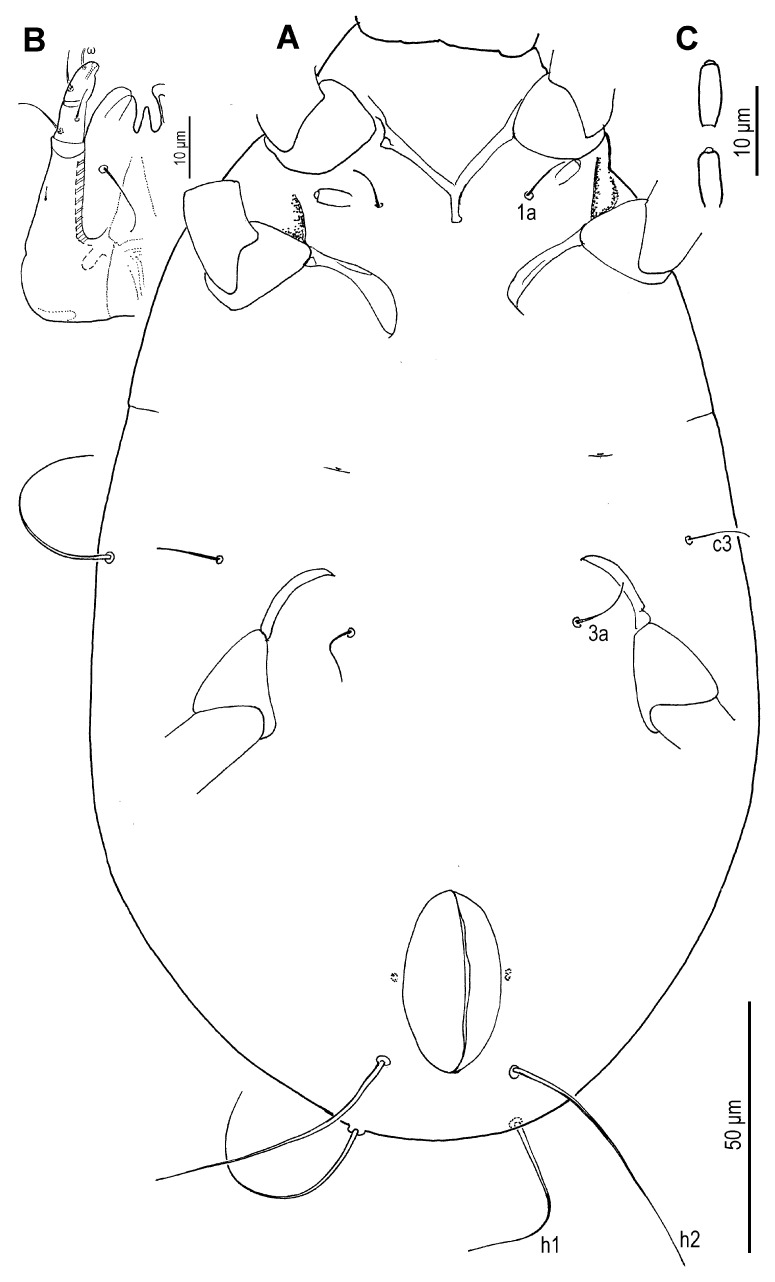
*Neosuidasia sjorsvandenbergi* sp. nov. (larva). (**A**). Idiosoma, ventral view; (**B**). Subcapitulum; (**C**). Claparède’s organ.

**Figure 25:**
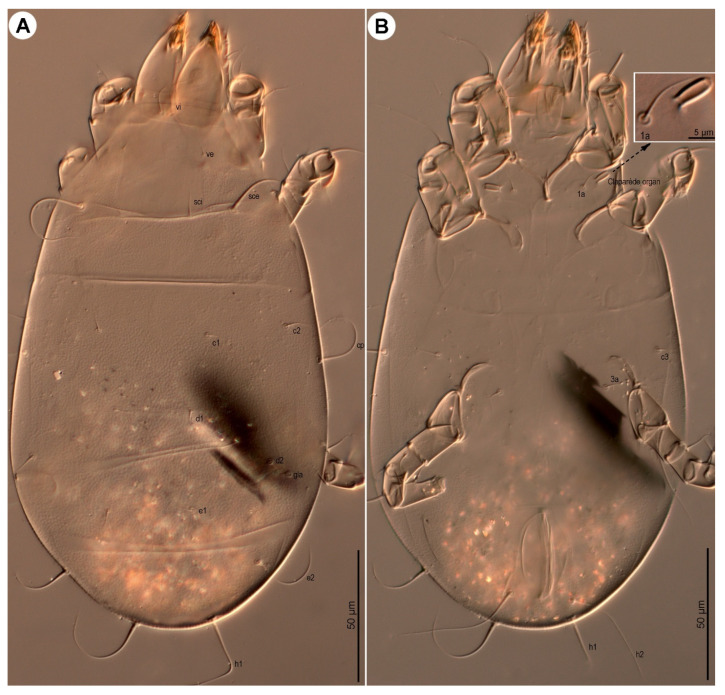
*Neosuidasia sjorsvandenbergi* sp. nov. (larva, DIC images). (**A**). Dorsal view; (**B**). Ventral view.

**Figure 26:**
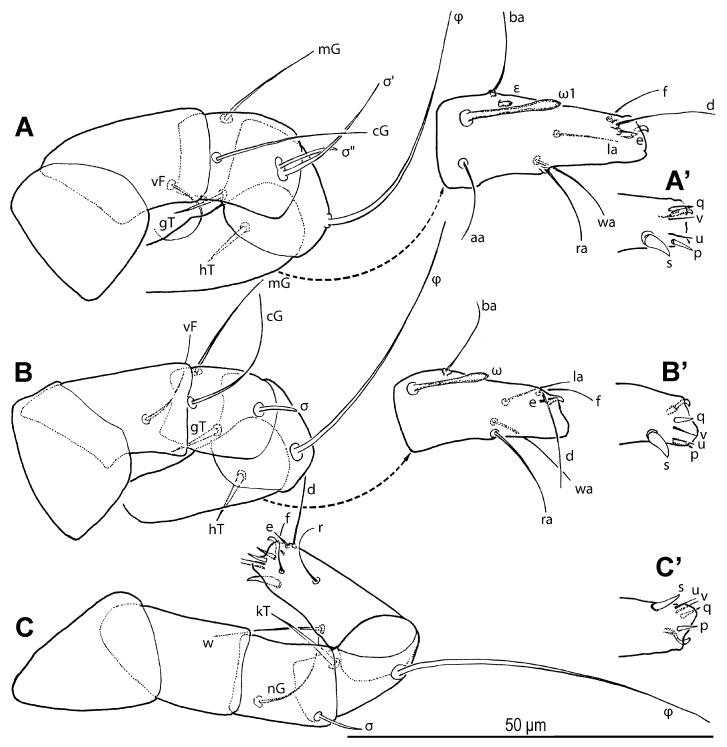
*Neosuidasia sjorsvandenbergi* sp. nov. (larva). (**A**–**C**). Legs I–III; (**A′**–**C′**). Pretarsi I–III, ventral view.

Larva (Figure 23, Figure 24, Figure 25 and Figure 26, Table 1)

**Dorsum** (Figure 23 and Figure 25A) oval; cuticle finely mammillated across most of dorsal surface, except for a narrow, smooth strip adjacent to sejugal suture. Prodorsal shield rectangular. Supracoxal sclerite and Grandjean’s organ as in the female; *scx* pectinate (Figure 23B), bearing 20–24 teeth. Idiosomal setae *f2* and *h3* absent; remaining setae filiform. Seta *vi* approximately 0.7× the width of the prodorsal shield; *ve* slightly more than 1/4 length of *vi*; *sci* roughly 1/5 length of *sce*; distance between *sci–sci* 1.6–1.7× that of *sci–sce*. Distance *gla*–*e2* nearly 4× *gla*–*d2*. Setae *c3* positioned anterolateral to coxae III, subequal in length to *c1*, *c2*, *d1*, *d2*, and *e1*; these setae notably distant from bases of the following row. Cupules indistinct.

Venter (Figure 24B,C and Figure 25B) with mammillated area as in tritonymph, finely mammillated across regions lateral to coxae III–IV, extending posterior beyond coxae IV and laterally to anus. Coxal apodemes and plates indistinct. Genital opening, coxisternal setae *4a*, *4b*, adanal and pseudanal setae absent. Claparède’s organ (Figure 24C) cylindrical (6.5–7.5), about twice as long as wide, located lateral to *1a* and posterior to trochanter I.

Gnathosoma (Figure 24B and Figure 25B): Structure and setation of the chelicerae, subcapitulum, and palps as in the female, differing only in dimensions (Table 1).

Legs (Figure 26): Leg II shorter than legs I and III; leg IV absent.

Leg I (Figure 26A,A′): Genual solenidia ratio *σ′:σ″* = 2.0. Tibial solenidion *φ* extending well beyond tarsal claw tip. Tarsus nearly 2.0× basal width. Solenidion *ω1* cylindrical with a prominent terminal head; *ε* slightly tapered apically, positioned clearly anterolateral to base of *ω1*; *ω2* and *ω3* absent. Setae *aa*, *ba*, *wa*, *ra*, *la*, *d*, *f* filiform; *e* setiform. Subunguinal seta (*s*), proral (*p*, *q*), and unguinal setae (*u*, *v*) as in adult female.

Leg II (Figure 26B,B′): Genual solenidion *σ* reaching one-third of tibial length; tibial solenidion *φ* extending well beyond tarsal claw tip. Tarsus 2.1× basal width. Solenidion *ω* cylindrical with a prominent terminal head. Setae *ba*, *wa*, *ra*, *la*, *d*, *f* filiform; *e* setiform. Subunguinal seta (*s*), proral (*p*, *q*), and unguinal setae (*u*, *v*) as in adult female.

Leg III (Figure 26C,C′): Genual solenidion σ reaching about one-fourth of tibial length; *φ* extending well beyond tarsal claw tip. Tarsus 2.1× basal width. Setae *w*, *r*, *d*, *f* filiform; *e* setiform. Subunguinal seta (*s*), proral (*p*, *q*), and unguinal setae (*u*, *v*) as in adult female.

Egg (Figure 27A,B).

Ovate in form, approximately twice as long (134–169 μm) as wide (78–99 μm); shell surface smooth, lacking distinct ornamentation.

Deutonymph (hypopus) unknown.

**Figure 27:**
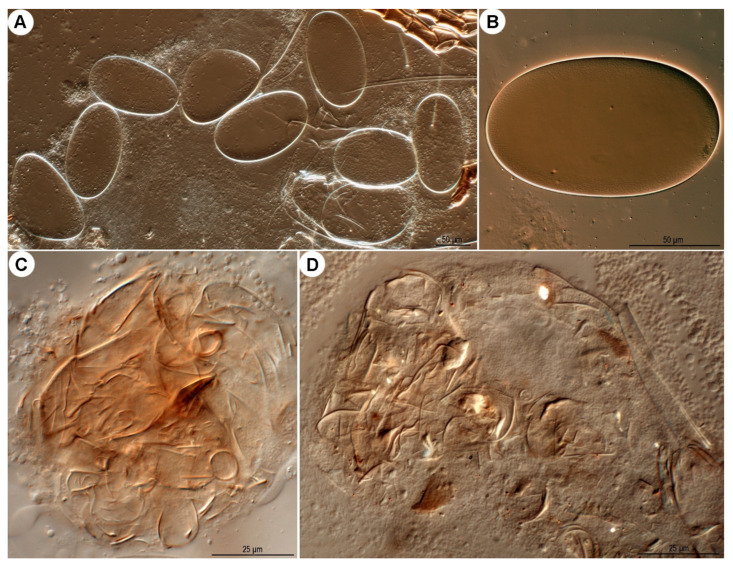
*Neosuidasia sjorsvandenbergi* sp. nov. (**A**,**B**). Eggs; (**C**,**D**). food boli.

**Etymology**

The new species *N. sjorsvandenbergi* is named in honour of Mr. Sjors van den Berg, (Quabio, the Netherlands) the provider of the type specimens.

**Biology**

Fragmented mite body parts and other unidentified materials were found in the food boli (Figure 27C,D), suggesting that this new species is likely detritivorous. The mite fragments are likely derived from shed exoskeletons of individuals in the mite population.

**Remarks**

The new species is morphologically similar to *N. faini* in the ornamentation of the idiosomal cuticle, setal proportions, and leg setation. However, it is readily distinguishable from *N. faini* by the following key traits.

**Key to species of *Neosuidasia***

1. Adult female: pseudanal seta *ps2* slightly longer than adanal seta *ad3*. Adult male: *ps2* less than twice as long as *ps3*. Genu I with *σ′* slightly longer than *σ″* in both sexes. ………………………………… *N. faini* Ranganath and ChannaBasavanna, 1983

- Adult female: pseudanal seta *ps2* approximately twice as long as adanal seta *ad3*. Adult male: *ps2* more than 4× length of *ps3*. Genu I with *σ′* 1.2–1.6× length of *σ″* in female, 1.5–1.9× in male. …………………… *N. sjorsvandenbergi* Fan & Faraji, sp. nov.

### 3.4. Ontogenetic Changes of Chaetotaxy

*Neosuidasia sjorsvandenbergi* sp. nov. undergoes four postembryonic stages: larva, protonymph, tritonymph, and adult. During the postlarval stages, specific setae are progressively added, while some existing setae change in position and/or length (Table 2). The larva bears a reduced complement of idiosomal setae. In the protonymph, the full set of dorsal idiosomal setae is established following the addition of two opisthosomal setae; pseudanal and genital setae also appear at this stage. Two coxisternal setae are added during the tritonymph stage. The adult male retains the same idiosomal setation as the tritonymph, whereas the adult female develops three adanal setae. Notably, setae *sci*, *c1*, *c2*, *d1*, *d2*, and *e1* tend to elongate with age, and setae *h1* and *h2* shift anteriorly in postlarval stages.

Palpal and gnathosomal chaetotaxy remains relatively stable across stages but vary slightly in size.

Leg chaetotaxy is more complex (Table 3). In the larval stage, leg IV is absent, however, the larva bears a complement of setae on femora, genua, and tibiae I–III, as well as on tarsi II–III. Two additional sensory setae on tarsus I are added sequentially: one in the protonymph and the other in the tritonymph. Trochanteral setae do not appear until the tritonymphal stage. Leg IV first appears in the protonymph with a reduced setal complement, and its full chaetotaxy is completed during the tritonymph.

**Table 2:** Development of idiosomal chaetotaxy in *Neosuidasia sjorsvandenbergi* sp. nov. (setae are indicated where they first appear).

	Dorsal Setae	Ventral Setae
Larva	*vi*, *ve*, *sci*, *sce*, *scx*, *c1*, *c2*, *cp*, *c3*, *d1*, *d2*, *e1*, *e2*, *h1*, *h2*	*1a*, *3a*
Protonymph	*f2*, *h3*	*g*, *ps1*, *ps2*, *ps3*
Tritonymph	-	*4a*, *4b*
Adult female	-	*ad1*, *ad2*, *ad3*
Adult male	-	-

**Table 3:** Development of leg chaetotaxy in *Neosuidasia sjorsvandenbergi* sp. nov. (setae are indicated where they first appear).

	Trochanters	Femora	Genua	Tibiae	Tarsi
**Leg I**					
Larva	-	*vF*	*cG*, *mG*, *σ′*, *σ″*	*gT*, *hT*, *φ*	*aa*, *ba*, *wa*, *la*, *ra*, *d*, *e*, *f*, *s*, *u*, *v*, *p*, *q*, *ɷ1*, *ɛ*
Protonymph	-	-	-	-	*ɷ2*
Tritonymph	*pR*	-	-	-	*ɷ3*
Adult		-	-	-	-
**Leg II**					
Larva	-	*vF*	*cG*, *mG*, *σ*	*gT*, *hT*, *φ*	*ba*, *wa*, *la*, *ra*, *d*, *e*, *f*, *s*, *u*, *v*, *p*, *q*, *ɷ*
Protonymph	-	-	-	-	-
Tritonymph	*pR*	-	-	-	-
Adult		-	-	-	-
**Leg III**					
Larva	-	-	*nG*, *σ*	*kT*, *φ*	*w*, *r*, *d*, *e*, *f*, *s*, *u*, *v*, *p*, *q*
Protonymph	-	-	-	-	-
Tritonymph	*sR*	-	-	-	-
Adult	-	-	-	-	-
**Leg IV**					
Larva	-	-	-	-	
Protonymph	-	-	-	-	*w*, *r*, *d*, *u*, *v*, *p*, *q*
Tritonymph	-	*wF*	-	*φ*	*e*, *f*, *s*
Adult	-	-	-	-	-

Key to life stages of *Neosuidasia sjorsvandenbergi* sp. nov. (Deutonymph stage unknown).

1. Four pairs of legs present; genital opening present with at least one pair of genital papillae present (Figure 2 and Figure 20B); hysterosomal setae *f2* and *h3* present (Figure 20B); Claparède’s organ absent. ………………………………………………… 2

- Three pairs of legs present; genital opening and genital papillae absent (Figure 24A); *f2* and *h3* absent (Figure 24A); Claparède’s organ present between coxae I and II (Figure 24A). …………………………………………………… larva

2. Two pairs of genital papillae present (Figure 15A); coxisternal setae *4a* and *4b* present (Figure 15A); tarsus I with solenidion *ω3* (Figure 18A); femur IV with a seta (Figure 18D); tibia IV with a solenidion (Figure 18D). …………………… 4

- One pair of genital papillae present (Figure 20A); coxisternal setae *4a* and *4b* absent (Figure 20A); tarsus I without *ω3* (Figure 22A); femur IV and tibia IV nude (Figure 22D). ………………………………………………………… protonymph

3. Genital opening inverted V-shaped (Figure 2); genital folds present; receptaculum seminis (spermatheca) (Figure 2 and Figure 5E) or aedeagus (Figure 9 and Figure 10D) present. ……………………………………………………………… (adult) … 5

- Genital opening a simple longitudinal slit (Figure 15A); genital folds absent; receptaculum seminis or aedeagus absent. ……………………………… tritonymph

4. Aedeagus present (Figure 9 and Figure 10D); receptaculum seminis absent; para-anal suckers (Figure 9) and tarsus IV suckers present (Figure 12D); genital opening positioned between trochanters IV (Figure 9); adanal setae absent (Figure 9). …… adult male

- Aedeagus absent; receptaculum seminis present (Figure 2 and Figure 3B); para-anal suckers and tarsus IV suckers absent (Figure 2 and Figure 6D); genital opening positioned between coxae III and IV (Figure 2); 3 pairs of adanal setae present (Figure 2 and Figure 3B). …………………………………………………………… adult female

## 4. Discussion

Members of *Neosuidasia* have previously been recorded from relatively restricted geographic areas, mainly within tropical and subtropical zones. The discovery of *N. sjorsvandenbergi* sp. nov. in the Netherlands offers new insight into the distribution and diversity of this little-known genus. The only other described species, *N. faini* Ranganath et ChannaBasavanna, has been recorded in the Afrotropical, Indomalayan, and Neotropical realms [9,10,11]. The occurrence of *N. sjorsvandenbergi* sp. nov. in a temperate European environment suggests a broader ecological range for the genus than previously recognized. This finding underscores the importance of continued acarological research, even in areas considered taxonomically well studied.

The new species fits well in the genus *Neosuidasia*, sharing key diagnostic traits with the known member of the genus, particularly in the structure of the gnathosoma, dorsal idiosomal setation, and leg chaetotaxy. However, it can be readily distinguished by the lengths of the pseudanal setae, especially *ps2*, which justifies its recognition as a distinct species. These differences are consistent and stable across multiple specimens and developmental stages, indicating taxonomic divergence rather than phenotypic variation or developmental plasticity.

The comparative lengths of the adanal and pseudanal setae surrounding the anus in females, as well as the pseudanal setae in males, are key diagnostic features distinguishing *N. sjorsvandenbergi* sp. nov. from *N. faini*. Ranganath et al. [9] provided an excellent description and high-quality illustrations of *N. faini*, capturing fine morphological details. However, a discrepancy exists between Figure 5 and the associated setal measurements. Although measurements were given for five of the six pairs of setae surrounding the female’s anus, the value for *ps2* was omitted. Based on Figure 5, *ps2* (labeled as *a2*) appears to be similar in length to *ad3* (as *a1*) and noticeably shorter than *ps1* (as *a6*), *ps3* (as *a2*), *ad1* (as *a5*), and *ad2* (as *a3*).

As with *N. faini* in *Neosuidasia* and species from the genus *Suidasia*, the deutonymphal stage (hypopus) of *N. sjorsvandenbergi* sp. nov. has not been observed, and these taxa may not develop the deutonymphal stage at all.

*Neosuidasia sjorsvandenbergi* sp. nov. was discovered in a home poultry environment, a habitat similar to those for *N. faini*, which has been recorded in poultry feed [9,11], pig feed [11], and a woodlouse breeding facility [10]. These findings suggest that *Neosuidasia* species may be ecologically associated with environments rich in organic matter, indicating a potentially saprophagous lifestyle. The consistent presence of *Neosuidasia* in such anthropogenic settings also suggests that members of this genus may be more widely distributed than currently recognized, with their apparent rarity likely attributed to insufficient research. These findings on *N. sjorsvandenbergi* sp. nov. underscore the need for further studies in Suidasiidae.

## Data Availability

The original contributions presented in this study are included in the article. Further inquiries can be directed to the corresponding author.
